# Association between PFAS and renal function indicators in cord blood of newborns: modifying effects of newborn sex and maternal factors

**DOI:** 10.1080/07853890.2026.2678686

**Published:** 2026-06-01

**Authors:** Wu Yan, Penghong Xu, Ying Xu, Wentao Yang, Qian Huang, Sabitina Mrisho Mzava, Francis Manyori Bigambo, Dandan Wang, Xu Wang

**Affiliations:** ^a^ Clinical Medical Research Center, Children’s Hospital of Nanjing Medical University, Nanjing, China; ^b^ Department of Emergency, Pediatric Intensive Care Unit, Children’s Hospital of Nanjing Medical University, Nanjing, China; ^c^ Department of Neonatology, Children’s Hospital of Nanjing Medical University, Nanjing, China; ^d^ Department of Endocrinology, Children’s Hospital of Nanjing Medical University, Nanjing, China; ^e^ Department of Obstetrics, The Second Affiliated Hospital of Nanjing Medical University, Nanjing, China; ^f^ School of Public Health, Nanjing Medical University, Nanjing, China;; ^g^ Department of Environmental and Occupational Health, Muhimbili University of Health and Allied Sciences, Dar es Salaam, Tanzania

**Keywords:** Cord blood sample, per- and polyfluoroalkyl substances, renal function indicators, newborns

## Abstract

**Background:**

Evidence on the associations between per- and polyfluoroalkyl substances (PFAS) exposure and renal function in newborns remains limited, particularly in cord blood studies. This retrospective cross-sectional study aimed to explore the associations between individual and mixed PFAS exposure with renal function indicators in the cord blood and to examine whether these associations were modified by newborn sex and maternal factors.

**Materials and methods:**

A total of 403 newborns with available cord blood samples were included. Six PFAS and renal function indicators were measured in cord blood. Multivariable linear regression, quantile-based g-computation (qgcomp), and Bayesian kernel machine regression (BKMR) models were used to evaluate the associations of individual and mixed PFAS exposure with blood urea nitrogen (BUN), creatinine (Cr), the BUN-to-Cr ratio (UCR), and estimated glomerular filtration rate (eGFR).

**Results:**

In multivariable linear regression analyses, perfluorobutanoic acid (PFBA) and perfluorononanoic acid (PFNA) were positively associated with BUN; PFBA, perfluorooctanoic acid (PFOA), and PFNA were positively associated with the UCR; and PFBA and perfluorohexanoic acid (PFHxA) were positively associated with eGFR. PFBA and PFHxA were inversely associated with Cr. Mixture analyses further showed that PFAS co-exposure was positively associated with BUN, UCR, and eGFR, but negatively associated with Cr. These associations were more pronounced among male newborns, those delivered vaginally, and those born to mothers aged ≤30 years with a normal pre-pregnancy body mass index.

**Conclusion:**

These findings suggest that prenatal co-exposure to PFAS, particularly PFBA, PFNA, PFOA, and PFHxA, may be associated with alterations in renal function indicators in newborns. The associations appeared to differ by newborn sex and maternal characteristics. Prospective longitudinal studies are needed to determine whether these early biomarker differences persist after birth and are related to later kidney outcomes.

## KEY MESSAGES

- Cord blood PFAS exposure was associated with several renal function indicators in newborns.
- Both individual and mixed PFAS exposure showed associations with renal function indicators, and these associations varied by newborn sex and maternal factors.
- Prospective longitudinal studies are needed to clarify whether these early biomarker changes persist after birth and predict later kidney outcomes.

## 1. Introduction

Impaired renal function (IRF) indicates significant pathological changes in the kidney and may impact the functioning of other organs and systems, leading to disruption of the body’s homeostasis [1]. The world prevalence of chronic kidney disease (CKD) is estimated to be 8–18% in adults [2] and 15–74.7 cases per million in children [3]. However, neonatal-specific prevalence data are limited. The incidence of neonatal end-stage renal disease is estimated to be 7.1 per million age-related population [4]. In China, the pediatric prevalence of IRF is estimated to be 0.25%. Although the prevalence seems to be lower, it was documented that children with IRF may reach 0.49 million [5].

Per- and polyfluoroalkyl substances (PFAS) are emerging organic contaminants that are widely used in the manufacturing of industrial and consumer products such as outdoor clothing, carpet production, nonstick pot coatings, fire extinguishing products, and food packaging [6]. Previous studies have linked PFAS with IRF [7–18]. Despite some inconsistent results, these studies mostly investigated the effect of PFAS in adults with or without some other underlying chronic diseases. Three studies assessed the impact of PFAS on renal function in adolescents [9,12,13], and only one study addressed the effect of PFAS on renal function in children [13], indicating further investigation in this area is needed.

Notably, reduced estimated glomerular filtration rate (eGFR) was associated with serum PFNA and PFHxS [14,18] as well as serum PFOA and PFOS [18]. Additionally, a negative relationship was found between perfluorodecanoic acid (PFDA) and eGFR, while N-methyl-perfluorooctane sulfonamido acetic acid (MeFOSAA) was positively associated with eGFR [14]. However, longitudinal studies have reported that decreased eGFR was the cause, not due to increased PFOA, indicating a reverse causation [7,13]. Most previous studies used peripheral blood to assess the effect of PFAS on renal function. Using cord blood sample is not only the best choice for evaluating chemical exposure in newborns as they give a clear picture of their serum concentration, particularly at birth [19,20], but also cord blood measurements at birth eliminate concerns about reverse causation present in adult studies [7,13], since the renal function indicators are measured simultaneously with PFAS exposure at a single time point in early life.

Evidence has shown that maternal factors such as maternal age, education, and body mass index (BMI) have been linked to PFAS concentrations in cord blood [20]. Specifically, studies have reported that PFAS regulates several metabolic pathways that show differences between sexes and maternal BMI [21–23]. Other studies have associated PFAS concentrations with delivery mode [24] and education levels [25,26]. The findings of these studies emphasize the need to consider sex, maternal education, BMI, and delivery mode when evaluating the impact of PFAS on health outcomes. However, little information is available on the influence of newborn sex and maternal factors on the relationship between PFAS and renal function indicators among newborn infants.

We aimed to explore the associations between individual and mixture exposure to PFAS and indicators of renal function in the cord blood of newborns, as well as to investigate the modifying effects of newborn sex and maternal factors such as maternal age, education, pre-pregnancy BMI, and delivery mode.

## 2. Materials and methods

### 2.1. Study participants

We conducted a retrospective cross-sectional study that included 403 mother-newborn pairs recruited at the Second Affiliated Hospital of Nanjing Medical University between February 2021 and September 2022. The inclusion criteria consisted of pregnant women who were residents of Nanjing and planned to deliver at the research hospital. The exclusion criteria included women who underwent assisted delivery, had twins or multiple births, or suffered from serious chronic diseases such as heart failure, renal failure, acquired immunodeficiency syndrome (AIDS), and cancer. This study was conducted under the principles of the Declaration of Helsinki and was approved by the Ethics Committee of the Second Affiliated Hospital of Nanjing Medical University (2023-KY-170-01). Written informed consent was obtained from the parents of all newborns at the time of the original sample collection.

### 2.2. Measurement of PFAS concentrations

Cord blood samples were collected from the umbilical cord at delivery and preserved at −80 °C until analysis. Six PFAS, including perfluorobutanoic acid (PFBA), perfluorohexanoic acid (PFHxA), perfluorooctanoic acid (PFOA), perfluorononanoic acid (PFNA), perfluorobutane sulfonate (PFBS), and perfluorooctane sulfonate (PFOS) were quantified using online solid phase extraction coupled to high-performance liquid chromatography-turbo ion spray ionization-tandem mass spectrometry (online SPE-HPLC-TIS-MS/MS) as explained in detail in a previous study [27]. Shortly, one aliquot of 20 µL of the mixed standard and 20 µL of the internal standard were added to cord serum samples. 100 µL of cord serum samples were added to the samples and mixed with 300 µL methyl tert-butyl ether (MTBE). The samples were vortexed for 15 min, and ultrasonic inspection was conducted for 20 min, and then 25 mg MgSO4 was added to the samples to prevent enzyme activity. Thereafter, the samples were vortexed for 30 s and isolated by centrifuging at 10,000 RCF for 15 min. The supernatants were allowed to dry completely by placing them gently under the vacuum drier at 35ᵒC. The samples were reconstituted by 50 µL (methanol: water = 20:80), vortexing for 20 s, and then moved into 0.2 µm filters at 10000 rpm for 1 min. Finally, the samples were transferred into an injection vial for LC-MS analysis (injection volume: 1 µL). The lower limit of quantitation (LOQs) for cord serum PFAS was as follows: PFBA = 0.16 ng/mL, PFHxA = 0.80 ng/mL, PFOA = 0.40 ng/mL, PFNA = 0.80 ng/mL, PFBS = 0.16 ng/mL, and PFOS = 0.16 ng/mL. Value below the LOQ wasere imputed as LOQ divided by the square root of 2.

### 2.3. Renal function test

Cord blood samples collected and preserved were analyzed using an automatic biochemical analyzer to detect serum blood urea nitrogen (BUN) and creatinine (Cr). We calculated the BUN-to-Cr ratio (UCR) (Formula 1) and estimated the glomerular filtration rate (eGFR) (Formula 2) [28].

[Formula 1] $$\text{UCR}=\frac{\text{Cord serum BUN}}{\text{Cord serum Cr}}$$

[Formula 2] $$\text{eGFR}\left(\text{ml}/\text{min}/1.{\text{73m}}^{2}\right)=k*\text{length}/\text{Cr}$$

Where k is a constant = 0.45 for a full-term newborn and 0.33 for premature infants. Length refers to the height of a newborn.

### 2.4. Covariates

A face-to-face questionnaire was administered to the newborn’s parents to gather information such as maternal age, pre-pregnancy weight and height, maternal education, passive smoking (detected by a question, does anyone in your household smoke? The response was either yes, or no), parity, delivery method, newborn sex, infant birth weight, infant birth height, gestational diabetes mellitus, and maternal hypertension. Covariates were selected based on previous literature [12,13] and visualized in the directed acyclic graph (DAG), as shown in Figure S1.

### 2.5. Statistical analysis

We performed a descriptive analysis to describe the participants’ characteristics. The results are presented as frequency (n) and percentage (%) for categorical variables and mean and standard deviation (SD) or median and interquartile range (IQR) for continuous variables. PFAS and renal function indicators were right-skewed and were log10-transformed.

Multivariable linear regression was used to explore the association between individual exposure to serum PFAS and renal function indicators in newborn infants. The models were adjusted for maternal age, pre-pregnancy BMI, maternal education, passive smoking, parity, delivery mode, infant sex, birth weight, birth height, gestational diabetes mellitus, intrauterine asphyxia, premature rupture of amniotic fluid, hypertension, and thyroid disease. Multivariable linear regression models were fitted after log_10_-transformation of both PFAS and renal function indicators to improve normality and stabilize variance. We checked the linear regression assumptions for all models. The residuals approximately followed a normal distribution (Shapiro–Wilk, *p*-value > 0.05), and the Breusch–Pagan test (*p*-value > 0.05) revealed no clear signs of heteroscedasticity. We evaluated multicollinearity using generalized variance inflation factors (GVIF); all values were below 2.0. Our findings suggest that there was no violation of the assumptions of linear regression. Moreover, the restricted cubic splines (RCS) curves were employed to evaluate a non-linear relationship between PFAS and renal function indicators using the package “plotRCS”.

In mixture analyses, quantile g-computation (qgcomp) was used to evaluate the mixture effects of six PFAS and renal function indicators. The qgcomp model estimates the effects of the mixture exposure by transforming the concentration of exposures into quantiles and then incorporating the weighted average of all exposures to generate the overall mixture effect estimates, interpreted as the mean difference in the outcome(s) across the quartile range of exposures. The bootstraps were set at 500 [29].

Moreover, we performed Bayesian kernel machine regression (BKMR) to investigate the association between PFAS mixture and renal function indicators. The BKMR model is a non-parametric approach used to assess the overall effects of environmental pollutant mixtures on health outcomes using a kernel function. The approach accounts for high-dimensional correlations, non-linear relationships, and interactions among environmental pollutants in the mixture. The detailed BKMR method is presented elsewhere [30]. In the current study, the BKMR was estimated by incorporating 50,000 iterations. The covariates adjusted in the models were maternal age, pre-pregnancy BMI, maternal education, passive smoking, parity, delivery mode, infant sex, birth weight, birth height, gestational diabetes mellitus, intrauterine asphyxia, premature rupture of amniotic fluid, hypertension, and thyroid disease.

The overall effect of the mixture of serum PFAS on renal function indices in newborn infants was estimated by comparing the difference when all PFAS were set at particular percentiles (25th to 75th) with their median value. We used a component-wise technique to give a posterior inclusion probability (PIP) to select relatively important individual components in the PFAS mixture. Furthermore, we assessed the bivariate interaction between each PFAS in the mixture with Cr, holding another PFAS at the 10th, 50th, and 90th percentiles and the remaining PFAS set to the median value (we presented the results for the overall population only).

We assessed the modifying effects of maternal factors such as maternal age, maternal education, pre-pregnancy BMI, mode of delivery, and newborn sex on the associations of PFAS with renal function indicators in newborn infants. Maternal age was grouped into ages ≤30 and >30 years. Pre-pregnancy BMI was categorized into normal weight (18–<24 kg/m^2^) and abnormal weight (<18 or ≥24 kg/m^2^). Maternal education was categorized into two groups: Below university education and university and above. All analyses were performed in all three models. All statistical analyses were performed in R version 4.0.3. The qgcomp and BKMR models were employed using the R packages “qgcomp” and “BKMR”, respectively. The significance was at *p* < 0.05.

## 3. Results

### 3.1. Baseline characteristics

Table 1 shows the characteristics of the study participants. A total of 403 newborn infants whose cord blood samples were available for detecting six PFAS and renal function indicators were recruited. Most of the participants were newborn males (54.3%). The mean maternal age was 29.65 years, the highest maternal education level was university and above (50.4%), and those who delivered vaginally were 57.1%. The median (IQR) for BUN, Cr, UCR, and eGFR were 10.08 (8.54, 11.76) mg/dL, 0.59 (0.52, 0.66) mg/dL, 17.39 (14.86, 20.43), and 38.25 (34.29, 43.24) ml/min/1.73 m2, respectively.

**Table 1.** Characteristics of newborn infants.

Characteristics	Overall (*N* = 403)
Maternal age (years)	29.65 ± 4.45
Pre-pregnancy weight (Kg)	57.59 ± 9.03
Height (cm)	162.04 ± 4.97
Pre-pregnancy BMI (Kg/m^2^)	21.91 ± 3.09
Maternal education	
Junior high school and below	61 (15.1)
High school/technical secondary school	34 (8.4)
Junior college	105 (26.1)
University and above	203 (50.4)
Passive smoking	
Yes	42 (10.4)
No	361 (89.6)
Parity	
1 Child	173 (42.9)
2 Children and above	230 (57.1)
Delivery mode	
Delivery vaginally	230 (57.1)
Cesarean section	173 (42.9)
Newborn sex	
Female	184 (45.7)
Male	219 (54.3)
Newborn birth weight (g)	3,382.51 ± 430.92
Newborn birth height (cm)	50.02 ± 1.13
Gestational diabetes mellitus	
Yes	55 (13.6)
No	348 (86.4)
Intrauterine asphyxia	
Yes	21 (5.2)
No	382 (94.8)
Premature rupture of amniotic fluid	
Yes	40 (9.9)
No	363 (90.1)
Maternal hypertension	
Yes	10 (2.5)
No	393 (97.5)
BUN (mg/dL)	10.08 [8.54, 11.76]
Cr (mg/dL)	0.59 [0.52, 0.66]
UCR	17.39 [14.86, 20.43]
eGFR (mL/min/1.73 m2)	38.25 [34.29, 43.24]

Note: BUN, blood urea nitrogen; Cr, creatinine; UCR, BUN-to-Cr ratio; eGFR, estimated glomerular filtration rate; BMI, Body mass index.

### 3.2. Distribution of PFAS concentrations

The highest and lowest detection rates of cord serum PFAS were 100% for PFBA and 50.6% for PFBS, respectively. PFBA also had the highest Geometric mean (GM) = 13.26, while PFBS had the lowest GM = 1.14 (Table S1). The bivariate correlation showed that PFBA and PFOA had the highest correlation (*r* = 0.63), and PFBS and PFOS had the lowest correlation (*r* = −0.01) (Figure S2).

### 3.3. Association between individual PFAS and renal function indicators

The adjusted linear regression showed that cord serum PFBA (β = 0.020, 95% CI: 0.004–0.035) and PFNA (β = 0.028, 95% CI: 0.009–0.047) were positively associated with BUN; PFBA (β = −0.011, 95% CI: −0.021 to −0.001) and PFHxA (β = −0.024, 95% CI: −0.042 to −0.005) were negatively associated with Cr; PFBA (β = 0.030, 95CI: 0.015-0.046), PFOA (β = 0.032, 95% CI: 0.009–0.055), and PFNA (β = 0.038, 95% CI: 0.019–0.057) were positively associated with UCR; and PFBA (β = 0.011, 95% CI: 0.001–0.021) and PFHxA (β = 0.024, 95% CI: 0.005–0.042) were positively associated with eGFR. No significant interaction effects were observed between PFAS with newborn sex, maternal age, maternal education, pre-pregnancy BMI, and delivery mode on renal function indicators, except PFOA with pre-pregnancy BMI on BUN and PFHxA with maternal education on UCR (Table 2).

**Table 2.** Association of PFAS and renal function indicators in cord blood of newborns (*N* = 403).

Outcomes	PFAS	β (95% CI)	*P*	P for interaction with				
				Newborn sex	Maternal age	Maternal education	Pre-pregnancy BMI	Delivery mode
BUN	PFBA	0.020 (0.004–0.035)	0.014	0.661	0.972	0.378	0.181	0.925
	PFHxA	−0.004 (−0.032 to 0.024)	0.793	0.899	0.290	0.136	0.343	0.381
	PFOA	0.020 (−0.003 to 0.042)	0.086	0.525	0.581	0.367	0.015	0.449
	PFNA	0.028 (0.009–0.047)	0.004	0.397	0.470	0.943	0.365	0.913
	PFBS	0.011 (−0.004 to 0.026)	0.145	0.705	0.093	0.658	0.184	0.890
	PFOS	−0.006 (−0.027 to 0.014)	0.551	0.676	0.774	0.993	0.840	0.645
Cr	PFBA	−0.011 (−0.021 to −0.001)	0.046	0.751	0.222	0.344	0.173	0.324
	PFHxA	−0.024 (−0.042, −0.005)	0.012	0.893	0.325	0.303	0.128	0.050
	PFOA	−0.012 (−0.027 to 0.003)	0.112	0.300	0.217	0.228	0.234	0.811
	PFNA	−0.010 (−0.023 to 0.002)	0.105	0.639	0.171	0.364	0.721	0.374
	PFBS	0.001 (−0.009 to 0.011)	0.861	0.471	0.113	0.782	0.374	0.898
	PFOS	0.001 (−0.013 to 0.015)	0.866	0.208	0.917	0.080	0.987	0.173
UCR	PFBA	0.030 (0.015–0.046)	<0.001	0.516	0.397	0.801	0.667	0.453
	PFHxA	0.020 (−0.008 to 0.048)	0.169	0.831	0.090	0.031	0.960	0.031
	PFOA	0.032 (0.009–0.055)	0.006	0.187	0.790	0.920	0.102	0.362
	PFNA	0.038 (0.019–0.057)	<0.001	0.594	0.847	0.498	0.505	0.482
	PFBS	0.010 (−0.005 to 0.026)	0.185	0.396	0.537	0.535	0.057	0.958
	PFOS	−0.007 (−0.028 to 0.013)	0.483	0.215	0.829	0.253	0.833	0.661
eGFR	PFBA	0.011 (0.003–0.021)	0.045	0.751	0.222	0.342	0.172	0.325
	PFHxA	0.024 (0.005–0.042)	0.012	0.895	0.325	0.308	0.129	0.050
	PFOA	0.012 (−0.003 to 0.027)	0.110	0.295	0.215	0.223	0.235	0.817
	PFNA	0.011 (−0.002 to 0.023)	0.102	0.648	0.170	0.374	0.716	0.378
	PFBS	−0.001 (−0.011 to 0.009)	0.865	0.468	0.111	0.776	0.374	0.898
	PFOS	−0.001 (−0.015 to 0.013)	0.872	0.209	0.921	0.080	0.973	0.172

Note: BUN, blood urea nitrogen; Cr, creatinine; UCR, BUN-to-Cr ratio; eGFR, estimated glomerular filtration rate. Statistical significance at *p* < 0.05.The model was adjusted for maternal age, pre-pregnancy BMI, maternal education, passive smoking, parity, delivery mode, infant sex, birth weight, birth height, gestational diabetes mellitus, intrauterine asphyxia, premature rupture of amniotic fluid, hypertension, and thyroid disease.

### 3.4. Association of the mixture of PFAS with renal function indicators using the qgcomp model

**Figure 1.**
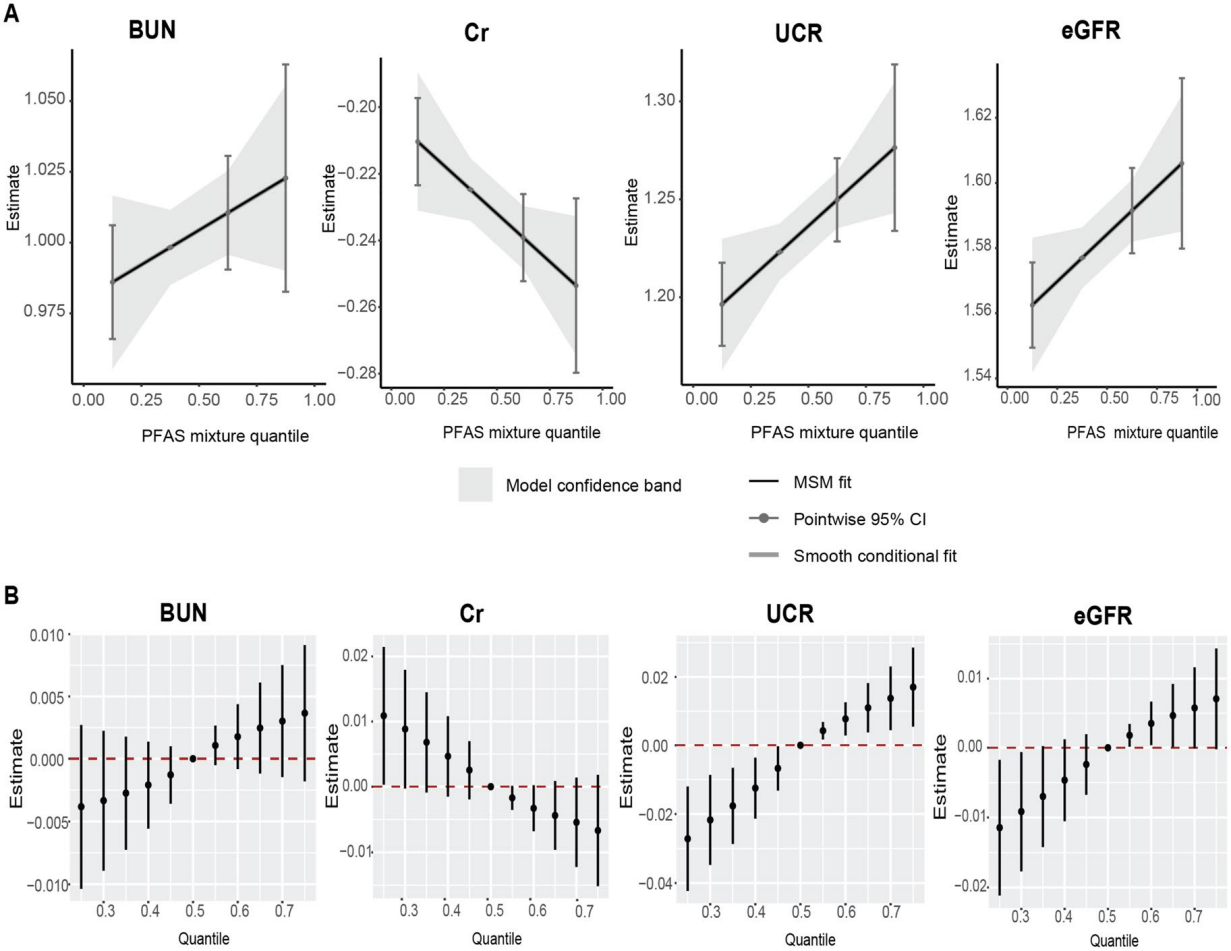
The overall effect of the mixture of serum per- and polyfluoroalkyl substances (PFAS) on renal function indicators in cord blood of newborns. (A) The quantile g-computation regression. (B) The Bayesian kernel machine regression (BKMR) models were estimated by comparing the difference when all PFAS were set at particular percentiles (25th to 75th) with their median. The models were adjusted for maternal age, pre-pregnancy body mass index (BMI), maternal education, passive smoking, parity, delivery mode, newborn sex, birth weight, birth height, gestational diabetes mellitus, intrauterine asphyxia, premature rupture of amniotic fluid, hypertension, and thyroid disease.

In the qgcomp model, the PFAS mixture was positively associated with UCR (β = 0.027, 95% CI: 0.007–0.047) and eGFR (β = 0.015, 95% CI: 0.001–0.028), and negatively associated with Cr (β = −0.015, 95% CI: −0.028 to −0.001) in the cord blood of newborns (Table S2 and Figure 1A).

### 3.5. Association of the mixture of PFAS with renal function indicators using the BKMR model

The BKMR model showed that the PFAS mixture was positively associated with BUN, UCR, and eGFR and negatively associated with Cr, consistent with the qgcomp except for BUN (Figure 1B). The PIPs of PFAS-renal function indicators are shown in Table S3. There was no evidence of interactions between PFAS and renal function indicators (Figures S3–S6).

### 3.6. Non-linear relationship between PFAS and renal function indicators using RCS

The RCS curves showed a non-linear, U-shaped relationship between PFOA and BUN (p for non-linear = 0.038) and UCR (p for non-linear = 0.003), suggesting that BUN and UCR were highest at both lower and higher levels of exposure. In addition, a non-linear, J-shaped relationship was observed between PFNA and UCR (p for non-linear =0.024), suggesting that there was little change in UCR at lower exposure levels, followed by a steep increase as the level of exposure increased (Figure S7).

### 3.7. Subgroup analyses of the association between PFAS and renal function indicators

#### 3.7.1. Newborn sex-specific analyses

In sex-specific analyses, the adjusted linear regression showed that PFBA and PFNA were positively associated with BUN in newborn male infants. PFBA and PFNA were positively associated with UCR in female and male infants, while PFOA was positively associated with UCR and eGFR only in newborn male infants (Table S4).

In the qgcomp model, the PFAS mixture was not associated with renal function indicators in newborn males and females (Table S5 and Figure S8A). In contrast, in the BKMR model, the PFAS mixture was positively associated with BUN, UCR, and eGFR and negatively associated with Cr in both newborn males and females (Figure S8B), indicating that sex did not modify the effect. The PIP of each PFAS-renal function indicator is presented in Table S6.

#### 3.7.2. Maternal age-specific analyses

In the maternal age-specific analyses, the linear regression model showed that PFBA and PFHxA were positively associated with UCR and eGFR, and PFOA and PFNA were positively associated with UCR. On the other hand, PFBA, PFHxA, and PFOS were negatively associated with Cr among newborns whose mothers’ ages were equal to or less than 30 years. PFNA was positively associated with BUN and UCR, and PFBS was positively associated with BUN and Cr, along with being negatively associated with eGFR among newborns whose mothers’ ages were greater than 30 years (Table S7).

**Figure 2.**
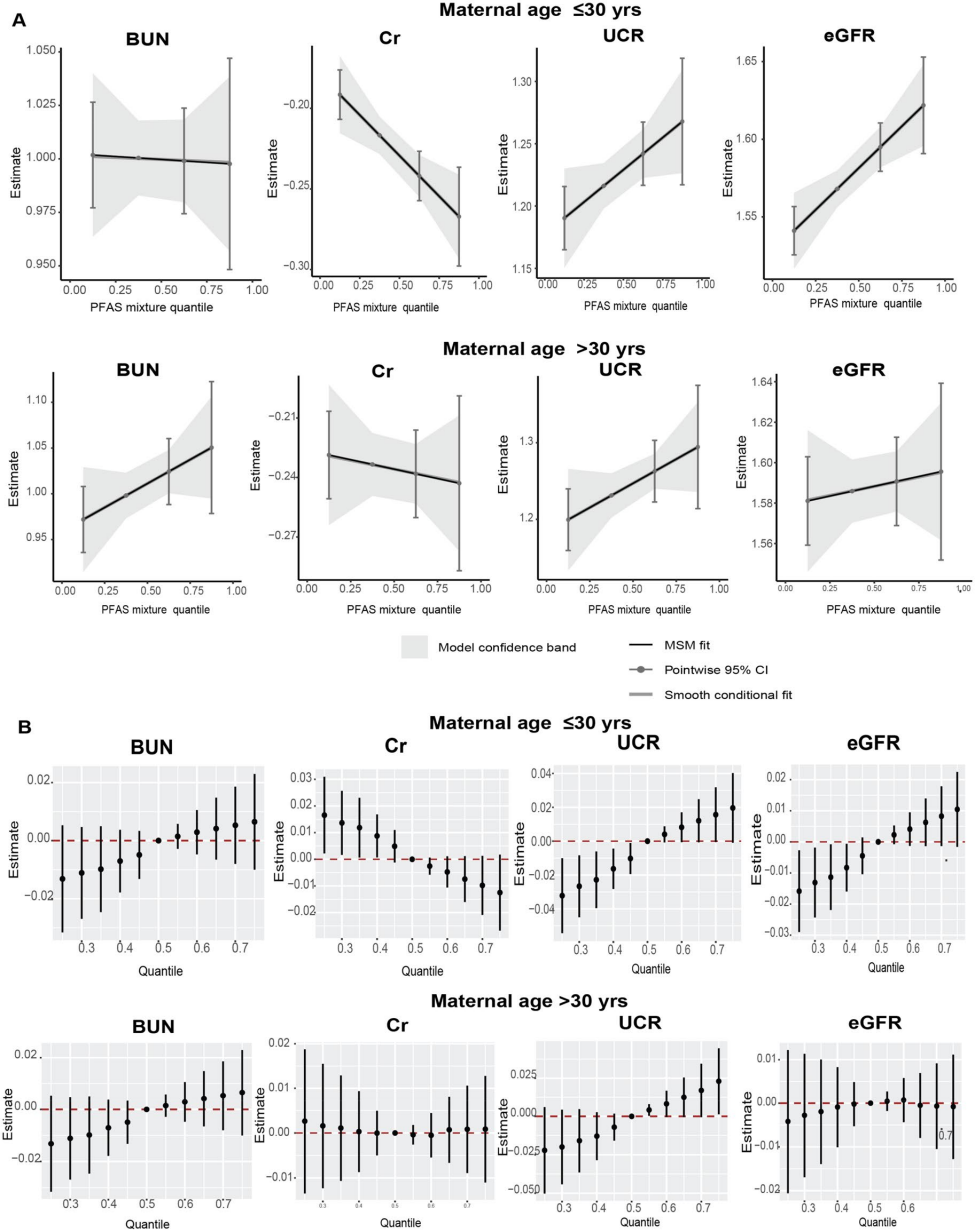
The overall effect of the mixture of serum per- and polyfluoroalkyl substances (PFAS) on renal function indicators in cord blood of newborns by maternal age. (A) The quantile g-computation regression. (B) The Bayesian kernel machine regression (BKMR) models were estimated by comparing the difference when all PFAS were set at particular percentiles (25th to 75th) with their median. The models were adjusted for pre-pregnancy body mass index (BMI), maternal education, passive smoking, parity, delivery mode, newborn sex, birth weight, birth height, gestational diabetes mellitus, intrauterine asphyxia, premature rupture of amniotic fluid, hypertension, and thyroid disease.

In the mixture analyses, the qgcomp and BKMR models showed that the PFAS mixture was positively associated with UCR and eGFR and negatively associated with Cr among newborns whose mothers’ ages were equal to or less than 30 years (Figure 2 and Table S5). The PIP of each PFAS-renal function indicator is presented in Table S6.

#### 3.7.3. Maternal education-specific analyses

In the individual exposure, the linear regression showed that PFBA was positively associated with BUN and UCR; PFHxA was positively associated with UCR and eGFR and negatively associated with Cr; PFNA was positively associated with UCR and eGFR and negatively associated with Cr among newborn infants whose mothers’ education was below university. On the other hand, PFBA and PFOA were positively associated with UCR and eGFR and negatively associated with Cr. PFNA was positively associated with BUN and UCR among newborn infants whose mothers’ education was university and above (Table S8).

In the mixture analyses, the qgcomp showed that the PFAS mixture was not associated with renal function indicators, regardless of maternal education, except in BUN, which revealed a significant positive association (Table S5 and Figure S9A). The BKMR results showed positive trends between PFAS mixture with BUN, UCR, and eGFR, while Cr revealed negative trends in both newborn infants whose mothers had education below university and university and above (Figure S9B). The PIP of each PFAS is presented in Table S6.

#### 3.7.4. Maternal pre-pregnancy BMI-specific analyses

In the maternal pre-pregnancy BMI-specific analyses, the adjusted linear regression showed significantly positive associations between PFBA with UCR, PFHxA with eGFR, and PFNA with BUN and UCR among newborn infants whose mother had a normal pre-pregnancy BMI, while PFHxA was significantly negatively associated with Cr. On the other hand, PFBA was significantly positively associated with UCR among newborn infants whose mothers had abnormal pre-pregnancy BMI (Table S9).

**Figure 3.**
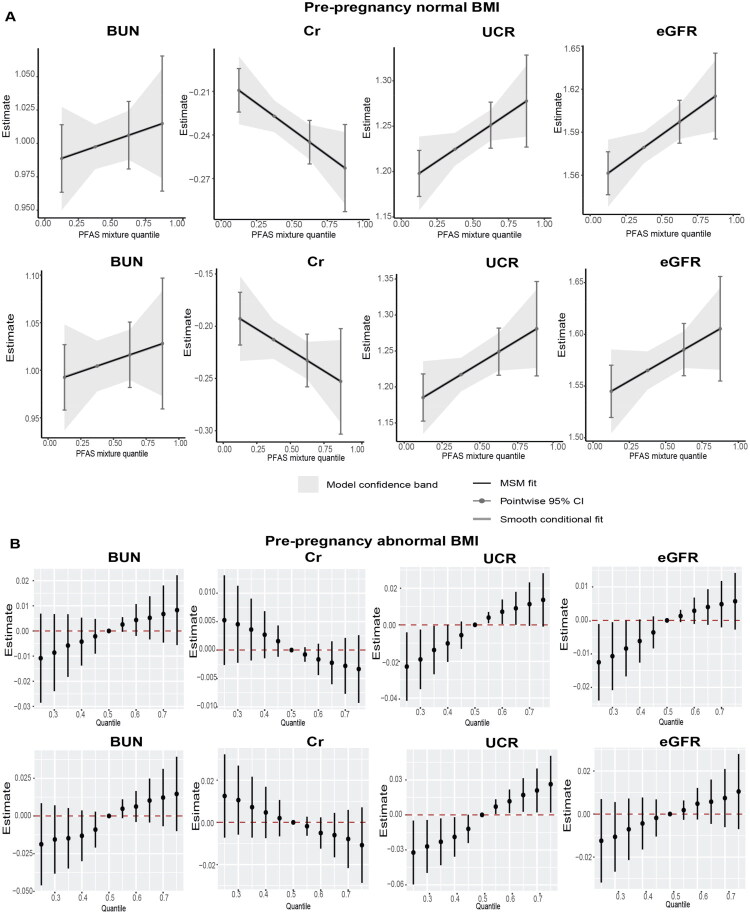
The overall effect of the mixture of serum per- and polyfluoroalkyl substances (PFAS) on renal function indicators in cord blood of newborns by pregnancy body mass index (BMI). (A) The quantile g-computation regression. (B) The Bayesian kernel machine regression (BKMR) models were estimated by comparing the difference when all PFAS were set at particular percentiles (25th to 75th) with their median. The models were adjusted for maternal age, maternal education, passive smoking, parity, delivery mode, newborn sex, birth weight, birth height, gestational diabetes mellitus, intrauterine asphyxia, premature rupture of amniotic fluid, hypertension, and thyroid disease.

In the qgcomp model, the PFAS mixture was positively associated with UCR and eGFR among newborn infants whose mothers had normal pre-pregnancy BMI, while the PFAS mixture was negatively associated with Cr (Figure 3A and Table S5). These association patterns were also observed in the BKMR Models in newborn infants (Figure 3B). The PIP of each PFAS-renal function indicator is presented in Table S6.

#### 3.7.5. Delivery mode-specific analyses

In individual exposure, the linear regression showed that PFBA, PFHxA, and PFNA were positively associated with UCR and eGFR, respectively; PFNA was positively associated with BUN; and PFBA and PFHxA were negatively associated with Cr among newborns delivered vaginally, while no significant associations were observed among newborns delivered through cesarean section (Table S10).

**Figure 4.**
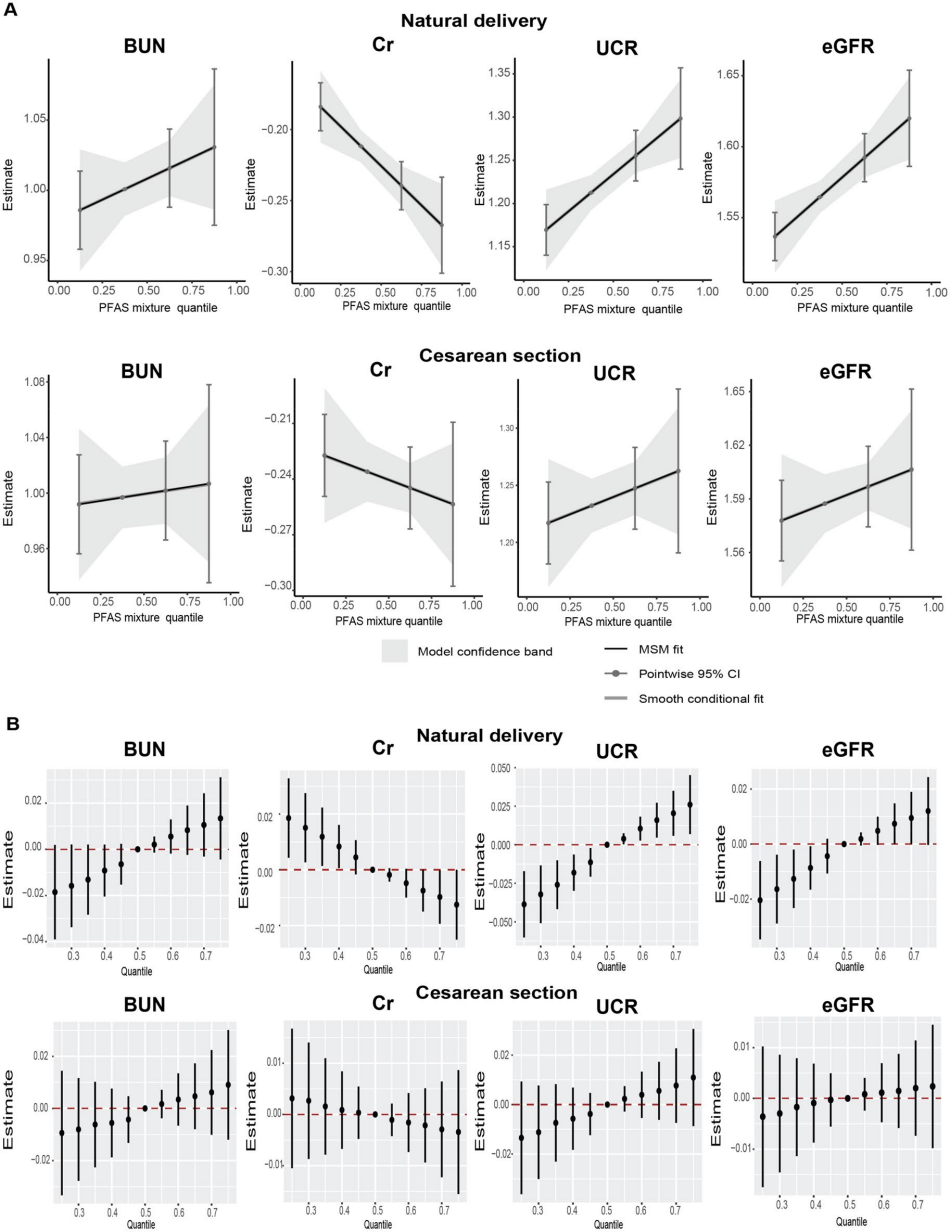
The overall effect of the mixture of serum per- and polyfluoroalkyl substances (PFAS) on renal function indicators in cord blood of newborns by delivery mode. (A) The quantile g-computation regression. (B) The Bayesian kernel machine regression (BKMR) models were estimated by comparing the difference when all PFAS were set at particular percentiles (25th to 75th) with their median. The models were adjusted for maternal age, pre-pregnancy body mass index (BMI), maternal education, passive smoking, parity, newborn sex, birth weight, birth height, gestational diabetes mellitus, intrauterine asphyxia, premature rupture of amniotic fluid, hypertension, and thyroid disease.

In the mixture analyses, the qgcomp showed that PFAS mixtures were positively associated with UCR and eGFR and negatively associated with Cr among newborn infants delivered vaginally but not by cesarean section (Figure 4A and Table S5). These associations were also present in the BKMR models (Figure 4B). The PIP of each PFAS is presented in Table S6.

## 4. Discussion

In this study, we explored the associations between individual and mixture exposures to PFAS and renal function indicators in the cord blood of newborns, and then investigated the modifying effects of newborn sex and maternal factors. We found that PFBA and PFNA were positively associated with BUN and UCR, while PFOA was positively associated with UCR, and PFBA and PFHxA were positively associated with eGFR in the cord blood of newborns. Conversely, PFBA and PFHxA were negatively associated with Cr. The qgcomp and BKMR models indicated that PFAS mixtures were positively associated with UCR and eGFR, and negatively associated with Cr. These associations were more evident in male newborns delivered vaginally by mothers aged 30 years or younger with a normal pre-pregnancy BMI.

Serum BUN is a product of protein metabolism, used as a subtle indicator of hemodynamic and renal perfusion changes and as an independent biomarker for poor clinical outcome prediction [31]. Serum Cr is a waste product carried in the blood until it is filtered by the kidneys and excreted in urine. BUN-to-Cr ratio (UCR) has recently been proposed as an independent predictor of deleterious clinical outcomes such as kidney injury [32], coronary artery disease [31], and ischemic stroke [33].

Our results showed that PFBA and PFNA were associated with increased BUN and UCR, and PFOA was associated with increased UCR in the cord blood of newborns. While PFBA and PFHxA were negatively associated with Cr in the cord blood of newborns. It is important to interpret these findings in the context of normal neonatal physiology rather than acute kidney injury (AKI) [34,35]. At birth, cord-blood BUN and Cr largely reflect transplacental equilibration with maternal levels and the immature state of fetal nephron function, not independent neonatal pathology, as umbilical cord urea and Cr closely mirror maternal concentrations and early neonatal values are strongly influenced by maternal kidney function and placental transfer [36,37].

Elevated BUN at this stage may reflect the newborn kidney’s limited capacity to concentrate urea or normal variations in protein and amino acid metabolism during the immediate postnatal period, consistent with studies in very preterm infants on parenteral nutrition where BUN primarily tracks amino acid and energy intake rather than intrinsic renal damage [38,39]. An elevated UCR may represent transient differences in urea handling relative to creatinine clearance in the setting of placental fetal dynamics, and in older populations, the UCR is known to be influenced by volume status and protein metabolism rather than being a specific marker of intrinsic renal injury [40].

Lower cord blood Cr likely reflects non‑pathological variation in determinants of neonatal Cr, such as maternal Cr, fetal muscle mass, gestational age, and early glomerular filtration, all of which are recognized influences on Cr based estimates of kidney function in newborns [41,42].

These patterns are consistent with normal physiological variation during the rapid maturation of renal function that occurs in the first days to weeks of life, when serum Cr levels generally decline while glomerular filtration rate increases and the neonatal kidneys progressively assume independent clearance [43,44].

Our findings do not include postnatal serial measurements or clinical AKI outcomes, so we do not interpret them as evidence of injury; rather, they suggest that prenatal PFAS exposure may subtly influence renal biomarker profiles during this critical developmental window [17,45].

Moreover, in the mixture effects, the PFAS mixture was negatively associated with eGFR [12,15,18]. Our results from the qgcomp and BMKR models consistently showed that PFAS mixtures were positively associated with UCR and eGFR and negatively associated with Cr in the cord blood of newborns. We also found a positive association between the PFAS mixture and BUN in the BKMR model, indicating that the BKMR model detected an additional association with BUN that was not observed in the qgcomp model. The discrepancies in the findings between the previous studies and ours may be attributed to variations in the study population, type of biospecimen used, and the time for collecting biospecimens.

In sex-specific analyses, we found that sex modified the individual effect of PFAS on renal function indicators, as more effects were more pronounced in newborn males than in females. However, this effect was not observed in the PFAS mixture, suggesting insufficient evidence of the modifying effect of newborn sex on the associations. Studies have highlighted that the effects of PFAS tend to be greater in males than in females [46–48]. The variation in PFAS concentrations between males and females might be linked to disparities in absorption, metabolism, distribution, excretion, and dietary needs between males and females [49].

In the present study, we found that maternal age can modify the association between PFAS and renal function indicators, with stronger effects observed among newborns whose mothers were ≤ 30 years old. Although the mechanisms underlying this pattern remain uncertain, previous epidemiologic studies and a recent systematic review have reported that maternal characteristics such as age are associated with differences in PFAS concentrations in pregnant women and neonates [25,50,51]. These findings indicate that maternal age can influence PFAS exposure and toxicokinetics, although the direction and magnitude of age-related differences are not fully consistent across studies.

Previous studies of persistent organic pollutants (POPs) have reported differences in serum concentrations by BMI, often with lower circulating levels of several lipophilic compounds at higher BMI, suggesting that greater adiposity can influence how these chemicals are distributed and diluted in the body [52,53]. Additionally, pre‑pregnancy BMI has been documented to modify the associations between PFAS exposure and maternal metabolic or hepatic outcomes. For example, Liao et al. [21] found that the relationship between prenatal PFAS exposure and maternal serum total bile acid levels differed by maternal pre‑pregnancy BMI, indicating that BMI can shape PFAS–health relationships. In our study, the association between PFAS and newborns’ renal function indicators was stronger in newborns born to mothers with normal pre‑pregnancy BMI than in those born to mothers with overweight or obesity. Consistent with this, a recent study of bisphenols reported that associations between bisphenol exposure and sex and thyroid hormone concentrations in cord blood varied by maternal pre‑pregnancy BMI, further supporting the plausibility of BMI‑related effect modification for endocrine‑disrupting chemicals in newborns [54]. However, a recent systematic review concluded that pre‑pregnancy BMI is generally not a strong or consistent determinant of PFAS concentrations in pregnant women or neonates, suggesting that the BMI‑related differences we observed may reflect more complex patterns involving body composition, exposure sources, and toxicokinetics, rather than a simple linear effect of BMI on PFAS concentrations [25].

The mode of delivery can influence exposure to PFAS. A previous study found that cord serum PFOS, PFOA, and PFNA were positively associated with natural delivery [24]. Our results showed that individual and mixture of PFAS were positively associated with BUN, UCR, and eGFR, and negatively associated with Cr among newborn infants delivered vaginally. The explanation can be that newborn infants born *via* cesarean section have reduced contact with maternal skin and vagina and perianal microbiota at birth [55], which can influence overall health and metabolic functions, possibly modulating how they process substances like PFAS. These proposed microbiota‑mediated pathways are speculative and should be viewed as hypotheses that require dedicated mechanistic research.

Moreover, education and income-to-poverty ratio have been reported to influence greater PFAS concentrations in maternal and newborn infants. This can be explained by the fact that women with higher incomes and advanced education can access and afford higher-priced products containing PFAS [25,26]. However, our results showed that PFAS was associated with renal function indices among newborn infants regardless of maternal education level, indicating that maternal education did not modify the association of PFAS and renal function indices among newborn infants.

Our research has the following strengths. First, we used cord blood samples to assess the effects of PFAS on renal function indices in newborn infants. A cord blood sample gives a clear picture of the serum concentration at the time of birth [19]. Second, we used statistical approaches such as qgcomp and BKMR and compared the findings, as each model has advantages and disadvantages. Third, we investigated the modifying effect of sex, maternal BMI, delivery mode, and education level on the relationship between PFAS and renal function among newborn infants.

This study has several limitations. First, a cross-sectional design cannot establish temporal relationships or causality. The possibility that renal function indicators influence PFAS retention (reverse causation) cannot be excluded, despite using cord blood. Second, PFAS exposure was measured only at birth. Maternal PFAS levels may change during pregnancy, and cord blood may not reflect cumulative prenatal exposure. Third, this study is a single-center recruitment. Generalizability to other populations (different PFAS exposure profiles, ethnicities, healthcare settings) is limited. Fourth, renal function biomarkers were measured, but no information is provided on clinical outcomes (e.g. subsequent diagnosis of renal disease, need for medical intervention). Therefore, the clinical significance of observed associations remains unclear. Fifth, despite extensive covariate adjustment, unmeasured confounders (e.g. maternal diet, maternal occupation, etc.) may influence results. Sixth, extensive subgroup analyses increase the risk of false-positive findings due to chance. Seventh, the detection limits for PFBS. Only 50.6% detection rate may limit the reliability of findings for this compound. Lastly, the biological mechanisms linking prenatal PFAS exposure to altered neonatal renal function indicators are not well-established. Further mechanistic research is needed.

## 5. Conclusions

This study found that PFBA and PFNA were positively associated with BUN and UCR, PFOA was positively associated with UCR, and PFBA and PFHxA were positively associated with eGFR in the cord blood of newborns. Contrarily, PFBA and PFHxA were negatively associated with Cr. Moreover, the PFAS mixture was associated with increased BUN, UCR, and eGFR and decreased Cr in the cord blood of newborns. These associations were stronger among newborn males and newborns whose mothers were 30 years or younger, had a normal pre-pregnancy BMI, and delivered vaginally. Prospective longitudinal studies are needed to determine whether these early biomarker differences persist after birth or are linked to later kidney outcomes.

## Supplementary Material

Supplementary materials.docx

## Data Availability

The data presented in this study are available on request from the corresponding author due to privacy or ethical restrictions.
