# Supplementary material for: Association between PFAS and renal function indicators in cord blood of newborns: modifying effects of newborn sex and maternal factors
Source: Ann Med. 2026 Jun 1;58(1):2678686. doi: 10.1080/07853890.2026.2678686 (PMC13228176; doi:10.1080/07853890.2026.2678686)
Supplement: Supplementary materials.docx [file IANN_A_2678686_SM3132.docx]

**Association between PFAS and renal function indicators in cord blood of newborns:** **Modifying effects of newborn sex and maternal factors**

**Contents**

**Supplementary Tables**

**Table S1.** Distribution of PFAS concentrations in newborns (N=403)

**Table S2.** Association of serum PFAS and renal function indicators in cord blood of newborns (N=403) based on quantile g-computation regression.

**Table S3.**The posterior inclusion probability (PIP) in the PFAS mixture-response function and renal function indicators in cord blood of newborns (N= 403)

**Table S4.** Association between cord serum PFAS and renal function indicators in cord blood of newborns stratified by newborns’ sex (N=403)

**Table S5.** Association of serum PFAS and renal function indicators in newborns based on quantile-based g-computation stratified by newborn’s sex and maternal factors (N=403)

**Table S6.** The posterior inclusion probability (PIP) in the PFAS mixture-response function and renal function indicators in cord blood of newborns stratified by newborn’s sex and maternal factors (N= 403)

**Table S7.** Association between cord serum PFAS and renal function indicators in cord blood of newborns stratified by maternal age (N=403)

**Table S8.** Association between serum PFAS and renal function indicators in cord blood of newborns (N=403) stratified by education levels

**Table S9.** Association between cord serum PFAS and renal function indicators in cord blood of newborns (N=403) stratified by Pre-pregnancy BMI

**Table S10.** Association between cord serum PFAS and renal function indicators in cord blood of newborns (N=403) stratified by delivery mode

**Supplementary Figures**

**Figure S1.** Shows a direct acyclic graph (DAG) for covariate selection

**Figure S2.** Spearman correlation between PFAS in cord blood of newborns. P >0.05

**Figure S3.** The Bivariate interaction between each per- and polyfluoroalkyl substance (PFAS) in the mixture with BUN, holding another PFAS at the 10th, 50th, and 90th percentiles and the remaining PFAS set to the median value. The Bayesian kernel machine regression (BKMR) models were adjusted for maternal age, pre-pregnancy body mass index (BMI), maternal education, passive smoking, parity, delivery mode, newborn sex, birth weight, and birth height, gestational diabetes mellitus, intrauterine asphyxia, premature rupture of amniotic fluid, hypertension, and thyroid disease.

**Figure S4.** Bivariate interaction between each per- and polyfluoroalkyl substance (PFAS) in the mixture with Cr, holding another PFAS at the 10th, 50th, and 90th percentiles and the remaining PFAS set to the median value. The Bayesian kernel machine regression (BKMR) models were adjusted for maternal age, pre-pregnancy body mass index (BMI), maternal education, passive smoking, parity, delivery mode, newborn sex, birth weight, birth height, gestational diabetes mellitus, intrauterine asphyxia, premature rupture of amniotic fluid, hypertension, and thyroid disease.

**Figure S5.** The bivariate interaction between each per- and polyfluoroalkyl substance (PFAS) in the mixture with UCR, holding another PFAS at the 10th, 50th, and 90th percentiles and the remaining PFAS set to the median value. The Bayesian kernel machine regression (BKMR) models were adjusted for maternal age, pre-pregnant body mass index (BMI), maternal education, passive smoking, parity, delivery mode, newborn sex, birth weight, and birth height, gestational diabetes mellitus, intrauterine asphyxia, premature rupture of amniotic fluid, hypertension, and thyroid disease.

**Figure S6.** The bivariate interaction between each per- and polyfluoroalkyl substance (PFAS) in the mixture with eGFR, holding another PFAS at the 10th, 50th, and 90th percentiles and the remaining PFAS set to the median value. The Bayesian kernel machine regression (BKMR) models were adjusted for maternal age, pre-pregnant body mass index (BMI), maternal education, passive smoking, parity, delivery mode, newborn sex, birth weight, and birth height, gestational diabetes mellitus, intrauterine asphyxia, premature rupture of amniotic fluid, hypertension, and thyroid disease.

**Figure S7.** Non-linear relationship between each of the PFAS and renal function indicators. Solid lines (blue) indicate beta coefficients and shaded areas indicate 95% confidence intervals. The modes were adjusted for maternal age, pre-pregnant body mass index (BMI), maternal education, passive smoking, parity, delivery mode, newborn sex, birth weight, birth height, gestational diabetes mellitus, intrauterine asphyxia, premature rupture of amniotic fluid, hypertension, and thyroid disease.

**Figure S8.** The overall effect of the mixture of per- and polyfluoroalkyl substances (PFAS) on renal function indicators in cord blood of newborns based on the newborn’s sex. A) The quantile g-computation regression. B) The Bayesian kernel machine regression (BKMR) models were estimated by comparing the difference when all PFAS were set at particular percentiles (25 to 75th) with their median. The models were adjusted for maternal age, pre-pregnancy body mass index (BMI), maternal education, passive smoking, parity, delivery mode, birth weight, birth height, gestational diabetes mellitus, intrauterine asphyxia, premature rupture of amniotic fluid, hypertension, and thyroid disease.

**Figure S9.** The overall effect of the mixture of serum per- and polyfluoroalkyl substances (PFAS) on renal function indicators in cord blood of newborns based on maternal education. A) The quantile g-computation regression. B) The Bayesian kernel machine regression (BKMR) models were estimated by comparing the difference when all PFAS were set at particular percentiles (25 to 75th) with their median. The models were adjusted for maternal age, pre-pregnancy body mass index (BMI), passive smoking, parity, delivery mode, newborn sex, birth weight, birth height, gestational diabetes mellitus, intrauterine asphyxia, premature rupture of amniotic fluid, hypertension, and thyroid disease.

**Supplementary Tables**

**Table S1.** Distribution of PFAS concentrations in newborns (N=403)

| PFAS (ng/mL) | LOQ | DR (%) | GM | GSD | Min | Percentile | | | | | Max |
| --- | --- | --- | --- | --- | --- | --- | --- | --- | --- | --- | --- |
|  |  |  |  |  |  | 5 | 25 | 50 | 75 | 95 |  |
| PFBA | 0.16 | 100.00 | 13.26 | 4.65 | 0.36 | 1.37 | 3.30 | 20.17 | 52.27 | 114.30 | 351.88 |
| PFHxA | 0.80 | 69.23 | 1.37 | 2.38 | 0.17 | 0.56 | 0.71 | 1.21 | 2.04 | 9.29 | 36.58 |
| PFOA | 0.40 | 94.54 | 9.29 | 2.94 | 0.28 | 0.28 | 5.97 | 10.71 | 19.20 | 31.45 | 71.46 |
| PFNA | 0.80 | 73.45 | 1.86 | 3.63 | 0.07 | 0.33 | 0.75 | 1.65 | 3.43 | 21.93 | 199.34 |
| PFBS | 0.16 | 50.62 | 0.19 | 5.05 | 0.002 | 0.02 | 0.09 | 0.16 | 0.33 | 2.55 | 1805.00 |
| PFOS | 0.16 | 99.26 | 3.09 | 3.26 | 0.09 | 0.33 | 1.48 | 3.24 | 6.73 | 21.15 | 65.09 |

Note: PFBA, perfluoro butanoic acid; PFHxA, perfluorohexanoic acid; PFOA, perfluorooctanoic acid; PFNA, perfluorononanoic acid; PFBS, perfluorobutanesulfonate; PFOS, perfluorooctane sulfonate; LOQ, limit of quantitation; DR, detection rate; GM, geometric mean; GSD, geometric standard deviation; Min, minimum; Max, maximum.

**Table S2.** Association of serum PFAS and renal function indicators in cord blood of newborns (N=403) based on quantile g-computation regression.

| Outcomes | PFAS mixture |  |  | Weight | | | | | | | | |
| --- | --- | --- | --- | --- | --- | --- | --- | --- | --- | --- | --- | --- |
|  | β (95%CI) | *P-*value |  | PFBA | PFHxA | | PFOA | | PFNA | PFBS | PFOS | |
| **Overall population** | | | | | | | | | | | | |
| BUN | 0.012 (-0.008, 0.032) | 0.232 |  | 0.247 | -0.610 | 0.157 | | | 0.472 | 0.123 | | -0.390 |
| Cr | -0.014 (-0.027, -0.001) | **0.032** |  | -0.088 | -0.410 | | | -0.014 | -0.273 | -0.003 | | -0.212 |
| UCR | 0.027 (0.005, 0.048) | **0.014** |  | 0.230 | 0.052 | | | 0.124 | 0.496 | 0.093 | | 0.006 |
| eGFR | 0.015 (0.001, 0.028) | **0.030** |  | 0.085 | 0.411 | | | 0.018 | 0.270 | 0.005 | | 0.211 |

Note: BUN, blood urea nitrogen; Cr, creatinine; UCR, BUN to Cr ratio; eGFR, estimated glomerular filtration rate.

The models were adjusted for maternal age, pre-pregnancy body mass index (BMI), maternal education, passive smoking, parity, delivery mode, newborn sex, birth weight, birth height, gestational diabetes mellitus, intrauterine asphyxia, premature rupture of amniotic fluid, hypertension, and thyroid disease. Statistical significance at *p* <0.05.

**Table S3.** The posterior inclusion probability (PIP) in the PFAS mixture-response function and renal function indicators in cord blood of newborns (N= 403)

| **PFAS** | **BUN** | **Cr** | **UCR** | **eGFR** |
| --- | --- | --- | --- | --- |
| PFBA | 0.576 | 0.315 | **0.100** | 0.097 |
| PFHxA | 0.544 | **0.614** | 0.075 | **0.415** |
| PFOA | 0.568 | 0.300 | 0.057 | 0.088 |
| PFNA | 0.595 | 0.**318** | **0.948** | **0.188** |
| PFBS | 0.552 | 0.132 | 0.023 | 0.006 |
| PFOS | 0.549 | 0.166 | 0.040 | 0.007 |

The model was adjusted for maternal age, pre-pregnancy BMI, maternal education, passive smoking, parity, delivery mode, newborn sex, birth weight, birth height, gestational diabetes mellitus, intrauterine asphyxia, premature rupture of amniotic fluid, hypertension, and thyroid disease.

**Table S4.** Association between cord serum PFAS and renal function indicators in cord blood of newborns stratified by newborns’ sex (N=403)

| PFAS | BUN | | Cr | | UCR | | eGFR | |
| --- | --- | --- | --- | --- | --- | --- | --- | --- |
|  | β (95% CI) | *p*-Value | β (95% CI) | *p*-Value | β (95% CI) | *p*-Value | β (95% CI) | *p*-Value |
| **Female** | | | | | | | | |
| PFBA | 0.016 (-0.008, 0.039) | 0.198 | -0.007 (-0.023, 0.009) | 0.419 | 0.022 (0.001, 0.044) | **0.043** | 0.007 (-0.009, 0.023) | 0.414 |
| PFHxA | 0.003 (-0.037, 0.043) | 0.882 | -0.021 (-0.048, 0.006) | 0.131 | 0.024 (-0.012, 0.060) | 0.199 | 0.021 (-0.006, 0.048) | 0.130 |
| PFOA | 0.006 (-0.027, 0.039) | 0.735 | -0.006 (-0.028, 0.016) | 0.595 | 0.012 (-0.018, 0.042) | 0.443 | 0.006 (-0.016, 0.028) | 0.593 |
| PFNA | 0.017 (-0.011, 0.044) | 0.231 | -0.013 (-0.032, 0.006) | 0.176 | 0.030 (0.005, 0.055) | **0.020** | 0.013 (-0.006, 0.032) | 0.175 |
| PFBS | 0.016 (-0.007, 0.038) | 0.168 | -0.004 (-0.019, 0.011) | 0.620 | 0.020 (-0.001, 0.040) | 0.060 | 0.004 (-0.011, 0.019) | 0.618 |
| PFOS | -0.012 (-0.043, 0.018) | 0.426 | 0.012 (-0.009, 0.032) | 0.267 | -0.024 (-0.052, 0.003) | 0.089 | 0.012 (-0.009, 0.032) | 0.267 |
| **Male** | | | | | | | | |
| PFBA | 0.024 (0.002, 0.045) | **0.034** | -0.013 (-0.028, 0.001) | 0.072 | 0.037 (0.014, 0.060) | **0.002** | 0.013 (-0.001, 0.028) | 0.071 |
| PFHxA | -0.006 (-0.046, 0.034) | 0.765 | -0.023 (-0.049, 0.003) | 0.086 | 0.017 (-0.027, 0.061) | 0.447 | 0.023 (-0.003, 0.050) | 0.084 |
| PFOA | 0.027 (-0.006, 0.059) | 0.108 | -0.021 (-0.043, -0.000) | 0.050 | 0.048 (0.013, 0.083) | **0.008** | 0.022 (0.0003, 0.043) | **0.049** |
| PFNA | 0.033 (0.006, 0.059) | **0.016** | -0.010 (-0.027, 0.008) | 0.290 | 0.042 (0.014, 0.071) | **0.004** | 0.010 (-0.008, 0.027) | 0.282 |
| PFBS | 0.006 (-0.015,0.028) | 0.563 | 0.006 (-0.008, 0.020) | 0.392 | 0.000 (-0.023, 0.023) | 0.989 | -0.006 (-0.020, 0.008) | 0.390 |
| PFOS | -0.001 (-0.030, 0.028) | 0.952 | -0.006 (-0.025, 0.013) | 0.547 | 0.005 (-0.027, 0.037) | 0.758 | -0.006 (-0.025, 0.013) | 0.547 |

Note: BUN, blood urea nitrogen; Cr, creatinine; UCR, BUN to Cr ratio; eGFR, estimated glomerular filtration rate.

The model was adjusted for maternal age, maternal education, passive smoking, parity, delivery mode, birth weight, birth height, gestational diabetes mellitus, intrauterine asphyxia, premature rupture of amniotic fluid, hypertension, and thyroid disease.

**Table S5.** Association of serum PFAS and renal function indicators in newborns based on quantile-based g-computation stratified by newborn’s sex and maternal factors (N=403)

| Subgroups | PFAS mixture |  |  | Weight | | | | | | | |
| --- | --- | --- | --- | --- | --- | --- | --- | --- | --- | --- | --- |
|  | β (95%CI) | *P-*value |  | PFBA | PFHxA | PFOA | PFNA | | PFBS | | PFOS |
| **Newborn sex** ^a^ | | | | | | | | | | |  |
| Female | | | | | | | | | | |  |
| BUN | 0.002 (-0.031, 0.036) | 0.888 |  | 0.512 | -0.326 | -0.317 | | 0.300 | | 0.188 | -0.357 |
| CR | -0.014 (-0.034, 0.006) | 0.173 |  | -0.110 | -0.463 | 0.003 | | -0.150 | | -0.233 | -0.045 |
| UCR | 0.016 (-0.014, 0.047) | 0.284 |  | 0.426 | 0.029 | -0.584 | | 0.293 | | 0.253 | -0.416 |
| eGFR | 0.014 (-0.006, 0.034) | 0.171 |  | 0.111 | 0.462 | -0.003 | | 0.149 | | 0.233 | 0.046 |
| Male | | |  |  | | | | | | | |
| BUN | 0.022 (-0.004, 0.049) | 0.098 |  | 0.263 | -0.002 | 0.311 | | 0.244 | | 0.094 | 0.089 |
| CR | -0.014 (-0.034, 0.007) | 0.164 |  | 0.895 | -0.247 | -0.415 | | -0.189 | | 0.105 | -0.148 |
| UCR | 0.016 (-0.014, 0.047) | 0.284 |  | 0.426 | 0.029 | -0.584 | | 0.293 | | 0.253 | -0.416 |
| eGFR | 0.014 (-0.006, 0.034) | 0.160 |  | -0.899 | 0.247 | 0.417 | | 0.188 | | -0.101 | 0.148 |
| **Maternal age ^b^** |  |  |  |  |  |  | |  | |  |  |
| **Maternal age ≤ 30 yrs** |  |  |  |  |  |  | |  | |  |  |
| BUN | -0.001(-0.026, 0.023) | 0.913 |  | 0.336 | 0.164 | 0.145 | | 0.519 | | -0.675 | -0.161 |
| Cr | -0.025(-0.041, -0.010) | **0.001** |  | -0.154 | -0.329 | -0.034 | | -0.074 | | -0.325 | -0.085 |
| UCR | 0.026(0.000, 0.051) | **0.047** |  | 0.322 | 0.232 | 0.131 | | 0.314 | | -0.641 | -0.359 |
| eGFR | 0.027(0.011, 0.042) | **0.001** |  | 0.150 | 0.322 | 0.064 | | 0.074 | | 0.304 | 0.087 |
| **Maternal age > 30 yrs** |  |  |  |  |  |  | |  | |  |  |
| BUN | 0.026 (-0.010 0.062) | 0.157 |  | -0.093 | -0.907 | 0.031 | | 0.533 | | 0.425 | 0.011 |
| Cr | -0.005 (-0.027, 0.017) | 0.674 |  | 0.435 | -0.317 | -0.273 | | -0.145 | | 0.565 | -0.265 |
| UCR | 0.032(-0.009, 0.072) | 0.126 |  | -0.801 | -0.199 | 0.136 | | 0.528 | | 0.192 | 0.144 |
| eGFR | 0.005 (-0.017, 0.027) | 0.665 |  | 0.435 | 0.320 | 0.271 | | 0.144 | | -0.565 | 0.266 |
| **Maternal education ^c^** | | | | | | | | | | | |
| Below university | | | | | | | | | | | |
| BUN | 0.026(-0.002, 0.054) | 0.068 |  | -0.693 | 0.134 | 0.332 | | 0.317 | | 0.217 | -0.307 |
| Cr | -0.011(-0.031, 0.010) | 0.301 |  | 0.293 | -0.376 | 0.701 | | -0.613 | | -0.012 | 0.005 |
| UCR | 0.038(0.006, 0.069) | **0.019** |  | -0.878 | 0.298 | 0.175 | | 0.504 | | 0.023 | -0.122 |
| eGFR | 0.011(-0.009, 0.032) | 0.273 |  | -0.274 | 0.384 | -0.726 | | 0.578 | | 0.023 | 0.015 |
| University and above |  |  |  |  |  |  | |  | |  |  |
| BUN | -0.004(-0.033, 0.026) | 0.801 |  | 0.447 | -0.497 | -0.412 | | 0.530 | | 0.023 | -0.091 |
| Cr | -0.017(-0.035, 0.001) | 0.067 |  | -0.125 | -0.273 | -0.423 | | 0.852 | | 0.148 | -0.178 |
| UCR | 0.015(-0.014, 0.044) | 0.320 |  | 0.514 | - 0.736 | 0.056 | | 0.326 | | - 0.264 | 0.104 |
| eGFR | 0.017(-0.001, 0.036) | 0.065 |  | 0.113 | 0.276 | 0.431 | | -0.817 | | -0.183 | 0.180 |
| **Pre-pregnant BMI ^d^** | | | | | | | | | | | |
| Normal weight | | | | | | | | | | | |
| BUN | 0.009 (-0.017, 0.034) | 0.500 |  | -0.255 | -0.270 | 0.599 | | 0.401 | | -0.062 | -0.413 |
| CR | -0.018(-0.033, -0.003) | **0.021** |  | -0.218 | -0.248 | 1.000 | | -0.110 | | -0.179 | -0.244 |
| UCR | 0.027(0.001, 0.052) | **0.041** |  | 0.045 | 0.061 | 0.357 | | 0.424 | | 0.113 | -1.000 |
| eGFR | 0.018(0.003, 0.033) | **0.020** |  | 0.213 | 0.251 | -1.000 | | 0.112 | | 0.180 | 0.245 |
| Abnormal weight | | | | | | | | | | | |
| BUN | 0.012(-0.023, 0.046) | 0.501 |  | 0.702 | 0.089 | -0.714 | | -0.189 | | 0.210 | -0.097 |
| Cr | -0.020(-0.045, 0.005) | 0.123 |  | 1.000 | -0.156 | -0.417 | | -0.198 | | -0.067 | -0.161 |
| UCR | 0.032(-0.001, 0.065) | 0.059 |  | 0.552 | 0.165 | -1.000 | | 0.023 | | 0.213 | 0.046 |
| eGFR | 0.020(-0.005, 0.045) | 0.121 |  | -1.000 | 0.155 | 0.413 | | 0.201 | | 0.067 | 0.165 |
| **Delivery mode e** | | | | | | | | | | | |
| Natural delivery | | | | | | | | | | | |
| BUN | 0.005(-0.031, 0.043) | 0.292 |  | 0.325 | -0.058 | 0.280 | | 0.225 | | 0.170 | -0.058 |
| Cr | -0.028(-0.045, -0.011) | **0.001** |  | 1.000 | - 0.482 | -0.189 | | -0.019 | | -0.187 | -0.123 |
| UCR | 0.043 (0.014, 0.072) | **0.004** |  | 0.129 | 0.325 | 0.233 | | 0.118 | | 0.194 | -1 |
| eGFR | 0.028(0.011, 0.045) | **0.002** |  | -1 | 0.4907 | 0.1941 | | 0.0316 | | 0.163 | 0.121 |
| Cesarean section | | | | | | | | | | | |
| BUN | 0.005(-0.031, 0.041) | 0.785 |  | -0.280 | -0.295 | -0.073 | | 0.891 | | 0.109 | -0.352 |
| CR | -0.009(-0.031, 0.013) | 0.438 |  | 0.684 | 0.132 | -0.335 | | -0.345 | | 0.185 | -0.320 |
| UCR | 0.015 (-0.021, 0.051) | 0.408 |  | -0.673 | -0.327 | 0.201 | | 0.735 | | 0.044 | 0.020 |
| eGFR | 0.010(-0.013, 0.032) | 0.408 |  | -0.740 | -0.098 | 0.360 | | 0.310 | | -0.162 | 0.330 |

Note: BUN, blood urea nitrogen; Cr, creatinine; UCR, BUN to Cr ratio; eGFR, estimated glomerular filtration rate. The models were adjusted for maternal age, pre-pregnancy body mass index (BMI), maternal education, passive smoking, parity, delivery mode, newborn sex, birth weight, birth height, gestational diabetes mellitus, intrauterine asphyxia, premature rupture of amniotic fluid, hypertension, and thyroid disease.

^a^ Newborn sex was not adjusted, ^b^ Maternal age, ^c^ Maternal education, ^d^ Pre-pregnant BMI was not adjusted, and ^e^ Delivery mode was not adjusted.

**Table S6.** The posterior inclusion probability (PIP) in the PFAS mixture-response function and renal function indicators in cord blood of newborns stratified by newborn’s sex and maternal factors (N= 403)

| **PFAS** | **BUN** | **Cr** | **UCR** | **eGFR** |
| --- | --- | --- | --- | --- |
| **Newborns’ sex** |  |  |  |  |
| **Female** |  |  |  |  |
| PFBA | 0.251 | 0.324 | 0.383 | 0.223 |
| PFHxA | 0.220 | **0.411** | 0.376 | 0.383 |
| PFOA | 0.230 | 0.330 | 0.330 | 0.267 |
| PFNA | 0.268 | 0.363 | 0.527 | 0.274 |
| PFBS | 0.245 | 0.264 | 0.462 | 0.207 |
| PFOS | 0.219 | 0.323 | 0.365 | 0.235 |
| **Male** |  |  |  |  |
| PFBA | 0.369 | 0.374 | 0.443 | 0.103 |
| PFHxA | 0.284 | 0.448 | 0.181 | 0.196 |
| PFOA | 0.274 | 0.429 | 0.212 | 0.169 |
| PFNA | 0.505 | 0.345 | 0.605 | 0.148 |
| PFBS | 0.162 | 0.266 | 0.091 | 0.035 |
| PFOS | 0.216 | 0.263 | 0.129 | 0.034 |
| **Maternal age** |  |  |  |  |
| **Maternal age ≤ 30 yrs** |  |  |  |  |
| PFBA | 0.151 | 0.479 | 0.535 | 0.302 |
| PFHxA | 0.218 | 0.649 | 0.488 | 0.595 |
| PFOA | 0.106 | 0.460 | 0.247 | 0.307 |
| PFNA | 0.231 | 0.373 | 0.445 | 0.194 |
| PFBS | 0.066 | 0.338 | 0.138 | 0.161 |
| PFOS | 0.122 | 0.357 | 0.165 | 0.125 |
| **Maternal age > 30 yrs** |  |  |  |  |
| PFBA | 0.431 | 0.191 | 0.356 | 0.081 |
| PFHxA | 0.535 | 0.314 | 0.360 | 0.176 |
| PFOA | 0.427 | 0.240 | 0.355 | 0.097 |
| PFNA | 0.818 | 0.267 | 0.759 | 0.140 |
| PFBS | 0.602 | 0.568 | 0.328 | 0.395 |
| PFOS | 0.374 | 0.211 | 0.270 | 0.092 |
| **Pre-pregnant BMI** |  |  |  |  |
| **Normal weight** |  |  |  |  |
| PFBA | 0.172 | 0.606 | 0.235 | 0.144 |
| PFHxA | 0.241 | 0.577 | 0.144 | 0.290 |
| PFOA | 0.186 | 0.549 | 0.145 | 0.080 |
| PFNA | 0.858 | 0.544 | 0.867 | 0.170 |
| PFBS | 0.137 | 0.528 | 0.094 | 0.013 |
| PFOS | 0.176 | 0.536 | 0.112 | 0.014 |
| **Abnormal weight** |  |  |  |  |
| PFBA | 0.370 | 0.348 | 0.513 | 0.186 |
| PFHxA | 0.313 | 0.423 | 0.368 | 0.355 |
| PFOA | 0.313 | 0.446 | 0.380 | 0.305 |
| PFNA | 0.274 | 0.425 | 0.367 | 0.265 |
| PFBS | 0.314 | 0.259 | 0.285 | 0.098 |
| PFOS | 0.299 | 0.326 | 0.308 | 0.180 |
| **Maternal education** |  |  |  |  |
| **Below University** |  |  |  |  |
| PFBA | 0.430 | 0.245 | 0.392 | 0.189 |
| PFHxA | 0.317 | 0.631 | 0.445 | 0.542 |
| PFOA | 0.339 | 0.285 | 0.236 | 0.240 |
| PFNA | 0.276 | 0.399 | 0.593 | 0.346 |
| PFBS | 0.220 | 0.181 | 0.170 | 0.128 |
| PFOS | 0.219 | 0.332 | 0.212 | 0.209 |
| **University and above** |  |  |  |  |
| PFBA | 0.217 | 0.474 | 0.401 | 0.238 |
| PFHxA | 0.259 | 0.412 | 0.223 | 0.245 |
| PFOA | 0.198 | 0.530 | 0.251 | 0.288 |
| PFNA | 0.457 | 0.333 | 0.465 | 0.104 |
| PFBS | 0.144 | 0.254 | 0.128 | 0.033 |
| PFOS | 0.169 | 0.334 | 0.161 | 0.087 |
| **Delivery mode** |  |  |  |  |
| **Natural delivery** |  |  |  |  |
| PFBA | 0.359 | 0.396 | 0.596 | 0.268 |
| PFHxA | 0.303 | 0.876 | 0.512 | 0.835 |
| PFOA | 0.346 | 0.349 | 0.341 | 0.192 |
| PFNA | 0.440 | 0.332 | 0.600 | 0.218 |
| PFBS | 0.254 | 0.249 | 0.264 | 0.126 |
| PFOS | 0.255 | 0.265 | 0.201 | 0.089 |
| **Cesarean section** |  |  |  |  |
| PFBA | 0.295 | 0.198 | 0.238 | 0.085 |
| PFHxA | 0.332 | 0.214 | 0.296 | 0.107 |
| PFOA | 0.260 | 0.407 | 0.187 | 0.305 |
| PFNA | 0.317 | 0.238 | 0.445 | 0.142 |
| PFBS | 0.218 | 0.146 | 0.126 | 0.039 |
| PFOS | 0.242 | 0.235 | 0.176 | 0.068 |

**Table S7.** Association between cord serum PFAS and renal function indicators in cord blood of newborns stratified by maternal age (N=403)

| PFAS | BUN | | Cr | | UCR | | eGFR | |
| --- | --- | --- | --- | --- | --- | --- | --- | --- |
|  | β (95% CI) | *p*-Value | β (95% CI) | *p*-Value | β (95% CI) | *p*-Value | β (95% CI) | *p*-Value |
| **Maternal age ≤ 30 yrs** | | | | | | | | |
| PFBA | 0.018(-0.002, 0.037) | 0.082 | -0.016(-0.029, -0.003) | **0.020** | 0.034(0.014, 0.053) | **0.001** | 0.016(0.003, 0.029) | **0.020** |
| PFHxA | 0.016(-0.021, 0.053) | 0.403 | -0.032(-0.057, -0.007) | **0.012** | 0.048(0.011, 0.086) | **0.012** | 0.032(0.007, 0.057) | **0.012** |
| PFOA | 0.015(-0.015, 0.045) | 0.328 | -0.018(-0.038, 0.002) | 0.083 | 0.033(0.003, 0.063) | **0.033** | 0.018(-0.002, 0.039) | 0.080 |
| PFNA | 0.020(-0.005, 0.046) | 0.113 | -0.016(-0.033,0.001) | 0.067 | 0.037(0.011, 0.062) | **0.005** | 0.016(-0.001, 0.033) | 0.064 |
| PFBS | 0.000(-0.019, 0.018) | 0.957 | -0.007(-0.019, 0.005) | 0.241 | 0.007(-0.011, 0.025) | 0.464 | 0.007(-0.005, 0.020) | 0.237 |
| PFOS | -0.006(-0.033, 0.022) | 0.682 | -0.003(-0.014, 0.022) | **0.038** | -0.010(-0.037, 0.018) | 0.490 | -0.004(-0.022,0.014) | 0.671 |
| **Maternal age > 30 yrs** | | | | | | | | |
| PFBA | 0.026(-0.002, 0.054) | 0.076 | 0.001(-0.017, 0.018) | 0.935 | 0.025(-0.003, 0.053) | 0.082 | -0.001(-0.018, 0.017) | 0.936 |
| PFHxA | -0.024(-0.068, 0.020) | 0.280 | -0.015(-0.042, 0.012) | 0.289 | -0.010(-0.053, 0.034) | 0.670 | 0.015(-0.012, 0.042) | 0.284 |
| PFOA | 0.028(-0.009, 0.065) | 0.140 | -0.005(-0.028, 0.018) | 0.666 | 0.033(-0.004, 0.070) | 0.079 | 0.005(-0.018, 0.028) | 0.668 |
| PFNA | 0.044(0.014, 0.075) | **0.005** | 0.002(-0.017, 0.022) | 0.827 | 0.042(0.012, 0.073) | **0.007** | -0.002(-0.022, 0.017) | 0.828 |
| PFBS | 0.036(0.006, 0.065) | **0.019** | 0.019(0.000, 0.037) | **0.047** | 0.017(-0.013, 0.047) | 0.266 | -0.019(-0.037, 0.000) | **0.047** |
| PFOS | -0.002(-0.037, 0.033) | 0.910 | -0.006(-0.027, 0.016) | 0.609 | 0.004(-0.031, 0.038) | 0.837 | 0.006(-0.016, 0.027) | 0.602 |

Note: BUN, blood urea nitrogen; Cr, creatinine; UCR, BUN to Cr ratio; eGFR, estimated glomerular filtration rate.

The model was adjusted for maternal education, passive smoking, parity, delivery mode, newborn sex, birth weight, birth height, gestational diabetes mellitus, intrauterine asphyxia, premature rupture of amniotic fluid, hypertension, and thyroid disease.

Statistical significance at *p* <0.05.

**Table S8.** Association between serum PFAS and renal function indicators in cord blood of newborns (N=403) stratified by education levels

| PFAS | BUN | | Cr | | UCR | | eGFR | |
| --- | --- | --- | --- | --- | --- | --- | --- | --- |
|  | β (95% CI) | *p*-Value | β (95% CI) | *p*-Value | β (95% CI) | *p*-Value | β (95% CI) | *p*-Value |
| **Below University** | | | | | | | | |
| PFBA | 0.023(0.002, 0.044) | **0.036** | -0.007( -0.022, 0.008) | 0.359 | 0.030 (0.008, 0.052) | **0.007** | 0.007( -0.008, 0.022) | 0.358 |
| PFHxA | 0.015( -0.025, 0.053) | 0.485 | -0.035(-0.063, -0.008) | **0.013** | 0.049(0.009, 0.089) | **0.017** | 0.035(0.008, 0.063) | **0.012** |
| PFOA | 0.029 (-0.009, 0.066) | 0.134 | -0.001( -0.028, 0.026) | 0.945 | 0.030(-0.009, 0.068) | 0.133 | 0.001( -0.026, 0.028) | 0.946 |
| PFNA | 0.023(-0.003, 0.050) | 0.090 | -0.019(-0.038, 0.000) | **0.053** | 0.042(0.015, 0.069) | **0.003** | 0.019(-0.0001, 0.038) | **0.053** |
| PFBS | 0.008 (-0.016, 0.031) | 0.510 | 0.003 ( -0.014, 0.019) | 0.762 | 0.003( -0.014, 0.019) | 0.762 | -0.003( -0.019, 0.014) | 0.759 |
| PFOS | -0.007(-0.036, 0.022) | 0.635 | 0.017( -0.004, 0.037) | 0.116 | -0.024 ( -0.054, 0.006) | 0.123 | -0.017(-0.037, 0.004) | 0.116 |
| **University and above** | | | | | | | | |
| PFBA | 0.013(-0.011, 0.037) | 0.281 | -0.016(-0.031, -0.001) | **0.042** | 2.862(0.005, 0.052) | **0.017** | 0.016(0.001, 0.031) | **0.041** |
| PFHxA | -0.020(-0.060, 0.020) | 0.335 | -0.016(-0.041, 0.010) | 0.231 | -0.004 (-0.045, 0.036) | 0.839 | 0.016 (-0.010, 0.042) | 0.224 |
| PFOA | 0.009(-0.020, 0.038) | 0.541 | -0.020 ( -0.038, -0.002) | **0.030** | 0.022(0.001, 0.058) | **0.045** | 0.020(0.002, 0.039) | **0.029** |
| PFNA | 0.028(0.001, 0.055) | **0.047** | -0.004 (-0.021, 0.014) | 0.679 | 0.031(0.004, 0.058) | **0.023** | 0.004(-0.014, 0.021) | 0.664 |
| PFBS | 0.010(-0.010, 0.030) | 0.322 | -0.0004 ( -0.013, 0.012) | 0.949 | -0.0004 (-0.013, 0.012) | 0.949 | 0.0005(-0.012, 0.013) | 0.944 |
| PFOS | -0.005 (-0.034, 0.025) | 0.753 | -0.011( -0.030, 0.008) | 0.259 | 0.006 (-0.023, 0.036) | 0.686 | 0.011 ( -0.008, 0.030) | 0.257 |

Note: BUN, blood urea nitrogen; Cr, creatinine; UCR, BUN to Cr ratio; eGFR, estimated glomerular filtration rate.

The model was adjusted for maternal age, pre-pregnancy BMI, passive smoking, parity, delivery mode, newborn sex, birth weight, birth height, gestational diabetes mellitus, intrauterine asphyxia, premature rupture of amniotic fluid, hypertension, and thyroid disease.

Statistical significance at *p* <0.05.

**Table S9.** Association between cord serum PFAS and renal function indicators in cord blood of newborns (N=403) stratified by Pre-pregnancy BMI

| PFAS | BUN | | Cr | | UCR | | eGFR | |
| --- | --- | --- | --- | --- | --- | --- | --- | --- |
|  | β (95% CI) | *p*-Value | β (95% CI) | *p*-Value | β (95% CI) | *p*-Value | β (95% CI) | *p*-Value |
| **Normal weight** | | | | | | | | |
| PFBA | 0.016 (-0.003, 0.034) | 0.095 | -0.010 (-0.022, 0.002) | 0.098 | 0.026(0.007, 0.045) | **0.007** | 0.010 ( -0.002, 0.022) | 0.098 |
| PFHxA | -0.004(-0.038, 0.029) | 0.790 | -0.024( -0.045, -0.002) | **0.034** | 0.019( -0.015, 0.053) | 0.274 | 0.024(0.002, 0.045) | **0.033** |
| PFOA | 0.014( -0.012, 0.040) | 0.303 | -0.009 (-0.027, 0.008) | 0.295 | 0.023( -0.004, 0.005) | 0.096 | 0.009 (-0.008, 0.027) | 0.292 |
| PFNA | 0.035(0.012, 0.058) | **0.004** | -0.005( -0.021, 0.010) | 0.503 | 0.040(0.016, 0.064) | **0.001** | 0.005 ( -0.010, 0.021) | 0.496 |
| PFBS | 0.005(-0.014, 0.024) | 0.607 | 0.003 ( -0.010, 1.521) | 0.690 | 0.002( -0.017, 0.022) | 0.810 | -0.003 (-0.015, 0.010) | 0.692 |
| PFOS | -0.009(-0.032, 0.015) | 0.478 | 0.001(-0.015, 0.016) | 0.935 | -0.009(-0.034, 0.015) | 0.462 | -0.001( -0.016, 0.015) | 0.937 |
| **Abnormal weight** | | | | | | | | |
| PFBA | 0.021(-0.011, 0.054) | 0.198 | -0.015(-0.037, 0.007) | 0.190 | 0.036 (0.006, 0.066) | **0.020** | 0.015( -0.007, 0.037) | 0.187 |
| PFHxA | -0.011(-0.070, 0.047) | 0.707 | -0.026 (-0.065, 0.014) | 0.208 | 0.014 (-0.041, 0.069) | 0.614 | 0.026( -0.014, 0.065) | 0.208 |
| PFOA | 0.012 (-0.038, 0.061) | 0.644 | -0.029 (-0.062, 0.005) | 0.094 | 0.040(-0.006, 0.087) | 0.089 | 0.029(-0.004, 0.062) | 0.092 |
| PFNA | 0.006 (-0.028, 0.039) | 0.750 | -0.020 (-0.043, 0.003) | 0.088 | 0.026 (-0.006, 0.057) | 0.118 | 0.020 (-0.003, 0.042) | 0.088 |
| PFBS | 0.022(-0.003, 0.048) | 0.093 | -0.001( -0.019, 0.017) | 0.914 | 0.023( -0.001, 0.048) | 0.063 | 0.001( -0.017, 0.019) | 0.911 |
| PFOS | -0.0003 (-0.045, 0.045) | 0.988 | -0.004(-0.034, 0.027) | 0.815 | 0.003(0.003, 0.046) | 0.879 | 0.004(-0.027, 0.034) | 0.808 |

Note: BUN, blood urea nitrogen; Cr, creatinine; UCR, BUN to Cr ratio; eGFR, estimated glomerular filtration rate. Statistical significance at *p* <0.05.

The model was adjusted for maternal age, maternal education, passive smoking, parity, delivery mode, newborn sex, birth weight, birth height, gestational diabetes mellitus, intrauterine asphyxia, premature rupture of amniotic fluid, hypertension, and thyroid disease.

**Table S10.** Association between cord serum PFAS and renal function indicators in cord blood of newborns (N=403) stratified by delivery mode

| PFAS | BUN | | Cr | | UCR | | eGFR | |
| --- | --- | --- | --- | --- | --- | --- | --- | --- |
|  | β (95% CI) | *p*-Value | β (95% CI) | *p*-Value | β (95% CI) | *p*-Value | β (95% CI) | *p*-Value |
| **Natural delivery** | | | | | | | | |
| PFBA | 0.021( -0.00001, 0.041) | 0.051 | -0.018 ( -0.032, -0.003) | **0.016** | 0.039 (0.017, 0.060) | **<0.001** | 0.018 (0.003, 0.032) | **0.016** |
| PFHxA | 0.010(-0.028, 0.0476) | 0.620 | -0.042( -0.068, -0.016) | **0.002** | 0.052 (0.013, 0.090) | **0.010** | 0.042(0.016, 0.068) | **0.002** |
| PFOA | 0.027(-0.007, 0.060) | 0.120 | -0.017 (-0.040, 0.006) | 0.156 | 0.043 (0.009, 0.078) | **0.014** | 0.017(-0.006, 0.040) | 0.155 |
| PFNA | 0.031 (0.005, 0.057) | **0.022** | -0.018(-0.037, -0.0002) | **0.049** | 0.049 (0.023, 0.076) | **<0.001** | 0.019 (0.0002, 0.037) | **0.049** |
| PFBS | 0.010 ( -1.001, 0.030) | 0.323 | -0.0003 (-0.014, 0.014) | 0.963 | 0.011 (-0.010, 0.032) | 0.324 | 0.0004(-0.014, 0.015) | 0.959 |
| PFOS | -0.004 ( -0.033, 0.023) | 0.727 | 0.009 (-0.011, 0.028) | 0.385 | -0.013( -0.042, 0.015) | 0.357 | -0.009 ( -0.028, 0.011) | 0.385 |
| **Cesarean Section** | | | | | | | | |
| PFBA | 0.013( -0.012, 0.039) | 0.311 | -0.004( -0.020, 0.012) | 0.597 | 0.017 (-0.007, 0.042) | 0.171 | 0.004 (-0.012, 0.020) | 0.593 |
| PFHxA | -0.021( -0.063, 0.021) | 0.329 | -0.002(-0.028, 0.025) | 0.888 | -0.019( -0.060, 0.022) | 0.367 | 0.002(-0.024, 0.028) | 0.882 |
| PFOA | 0.027 (-0.007, 0.060) | 0.120 | -0.017( -0.037, 0.003) | 0.102 | 0.019 ( -0.012, 0.051) | 0.226 | 0.017( -0.003, 0.037) | 0.100 |
| PFNA | 0.021 (-0.007, 0.050) | 0.144 | -0.005(-0.023, 0.013) | 0.554 | 0.027(-0.001, 0.055) | 0.062 | 0.006 (-0.012, 0.024) | 0.545 |
| PFBS | 0.014 (-0.009, 0.037) | 0.242 | 0.001(-0.014, 0.016) | 0.874 | 0.011( -0.010, 0.032) | 0.324 | -0.001(-0.016, 0.014) | 0.878 |
| PFOS | -0.012( -0.045, 0.020) | 0.457 | -0.011( -0.031, 0.009) | 0.286 | -0.001( -0.033, 0.031) | 0.940 | 0.011( -0.009, 0.032) | 0.283 |

Note: BUN, blood urea nitrogen; Cr, creatinine; UCR, BUN to Cr ratio; eGFR, estimated glomerular filtration rate.

The model was adjusted for maternal age, pre-pregnancy BMI, maternal education, passive smoking, parity, newborn sex, birth weight, birth height, gestational diabetes mellitus, intrauterine asphyxia, premature rupture of amniotic fluid, hypertension, and thyroid disease.

Statistical significance at *p* <0.05.

**
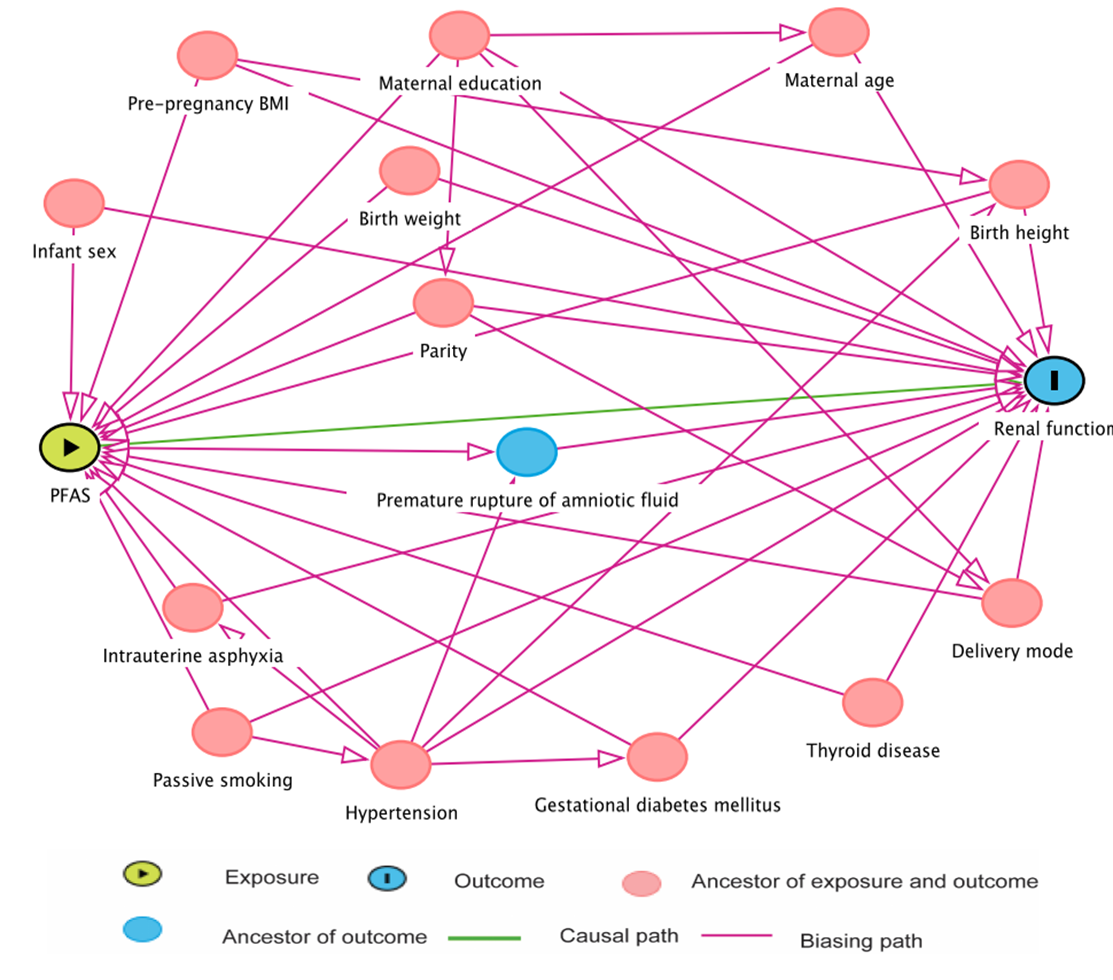
**

**Figure S1.** Shows a direct acyclic graph (DAG) for covariate selection

**
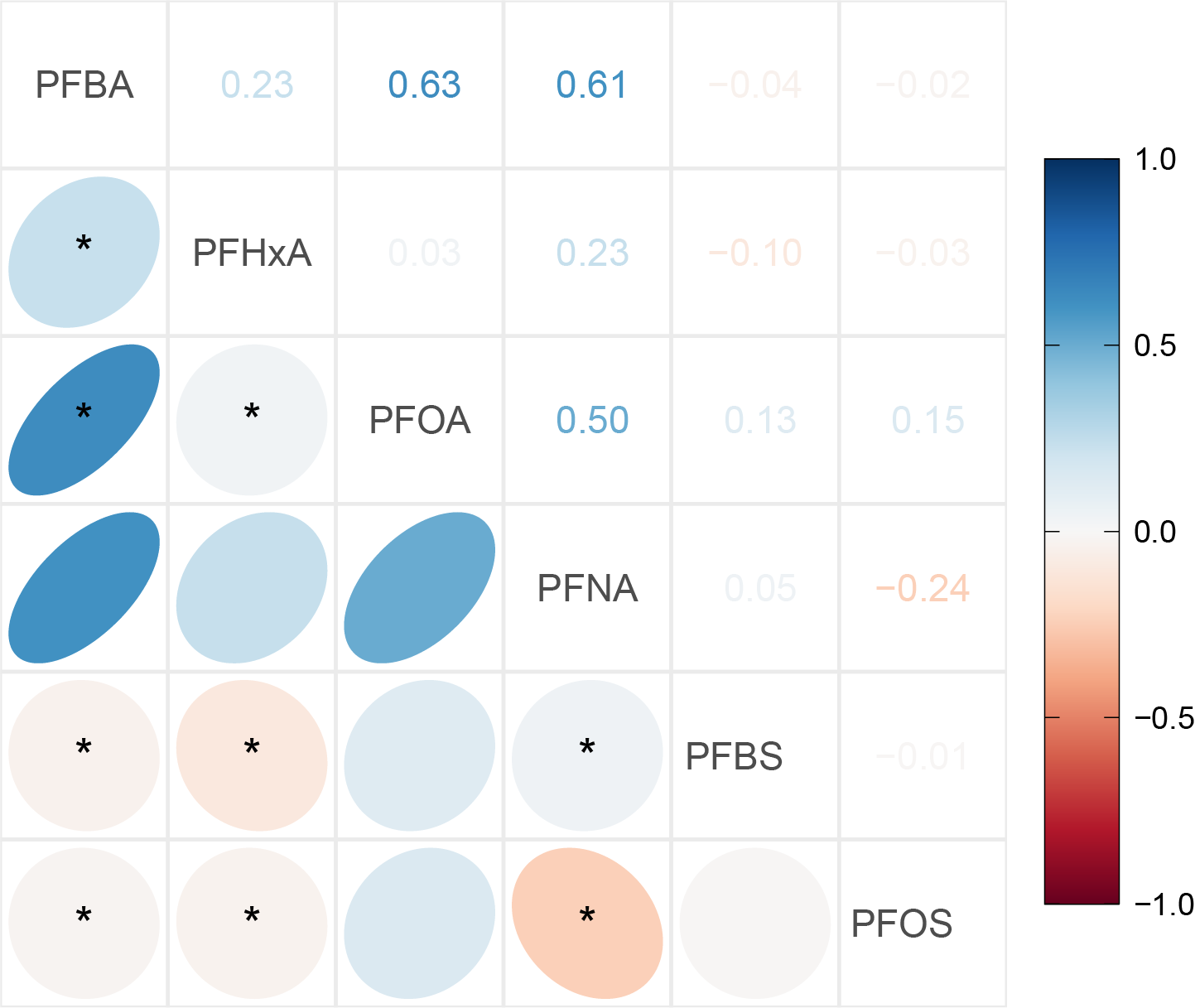
**

**Figure S2.** Spearman correlation between PFAS in cord blood of newborns. P >0.05


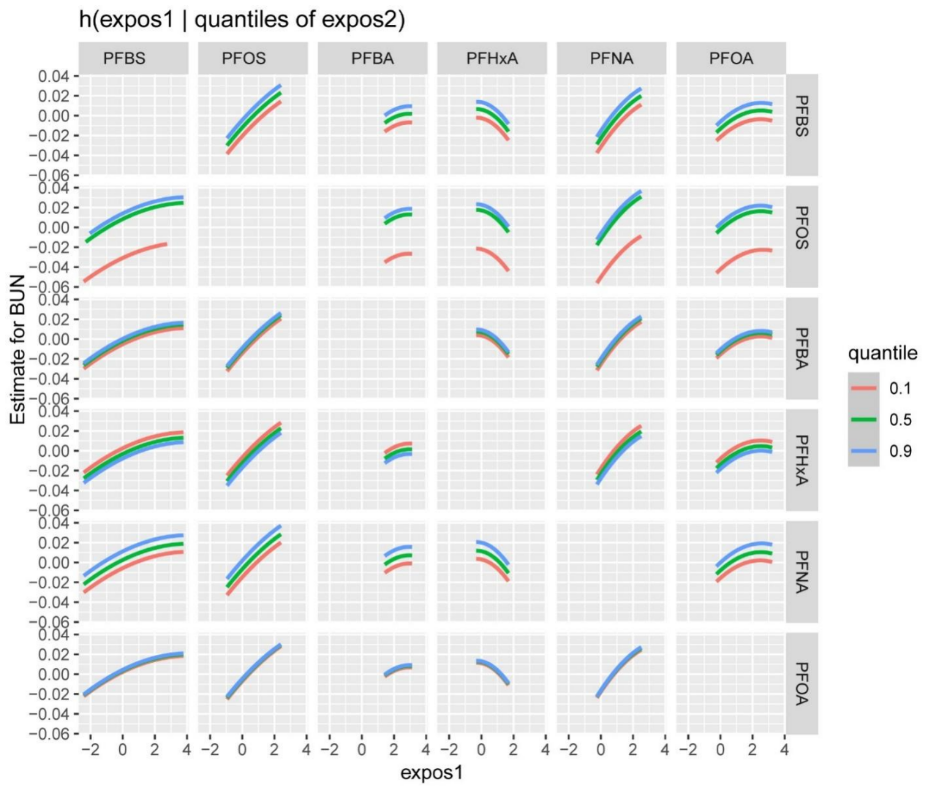


**Figure S3.** The Bivariate interaction between each per- and polyfluoroalkyl substance (PFAS) in the mixture with BUN, holding another PFAS at the 10th, 50th, and 90th percentiles and the remaining PFAS set to the median value. The Bayesian kernel machine regression (BKMR) models were adjusted for maternal age, pre-pregnancy body mass index (BMI), maternal education, passive smoking, parity, delivery mode, newborn sex, birth weight, and birth height, gestational diabetes mellitus, intrauterine asphyxia, premature rupture of amniotic fluid, hypertension, and thyroid disease.


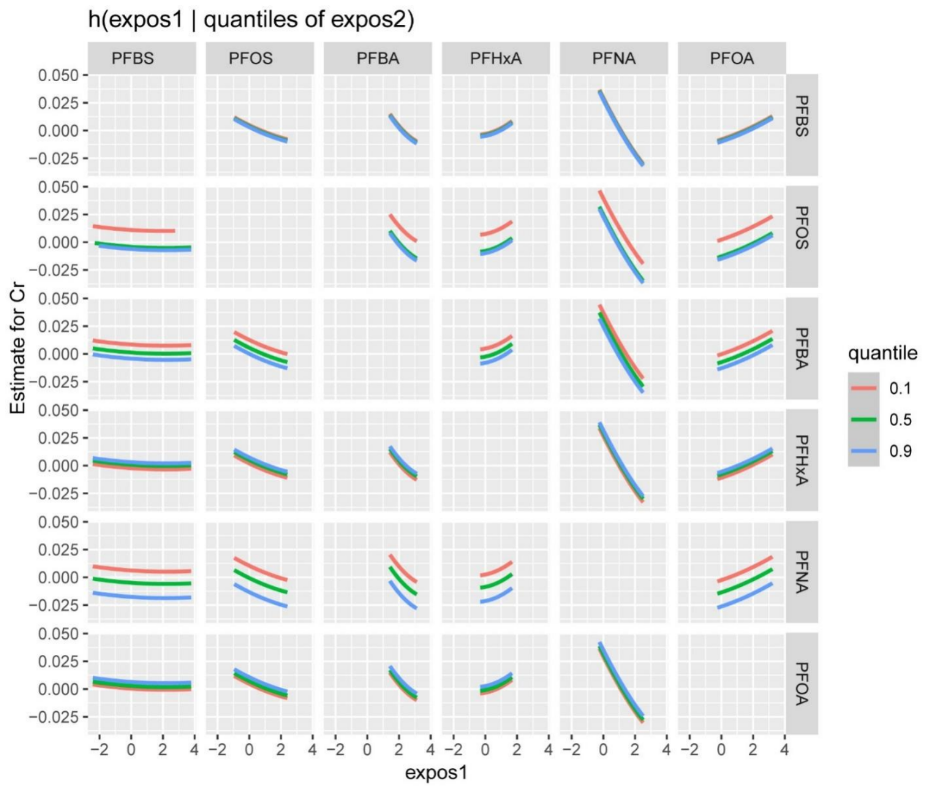
**Figure S4.** Bivariate interaction between each per- and polyfluoroalkyl substance (PFAS) in the mixture with Cr, holding another PFAS at the 10th, 50th, and 90th percentiles and the remaining PFAS set to the median value. The Bayesian kernel machine regression (BKMR) models were adjusted for maternal age, pre-pregnancy body mass index (BMI), maternal education, passive smoking, parity, delivery mode, newborn sex, birth weight, birth height, gestational diabetes mellitus, intrauterine asphyxia, premature rupture of amniotic fluid, hypertension, and thyroid disease.


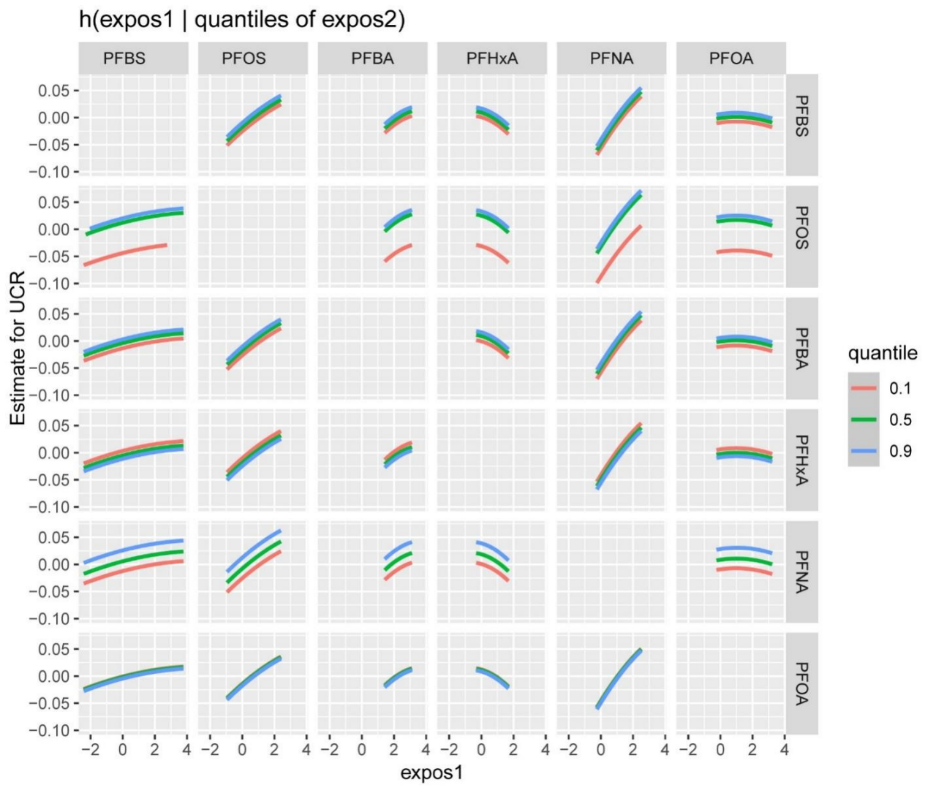


**Figure S5.** The bivariate interaction between each per- and polyfluoroalkyl substance (PFAS) in the mixture with UCR, holding another PFAS at the 10th, 50th, and 90th percentiles and the remaining PFAS set to the median value. The Bayesian kernel machine regression (BKMR) models were adjusted for maternal age, pre-pregnant body mass index (BMI), maternal education, passive smoking, parity, delivery mode, newborn sex, birth weight, and birth height, gestational diabetes mellitus, intrauterine asphyxia, premature rupture of amniotic fluid, hypertension, and thyroid disease.


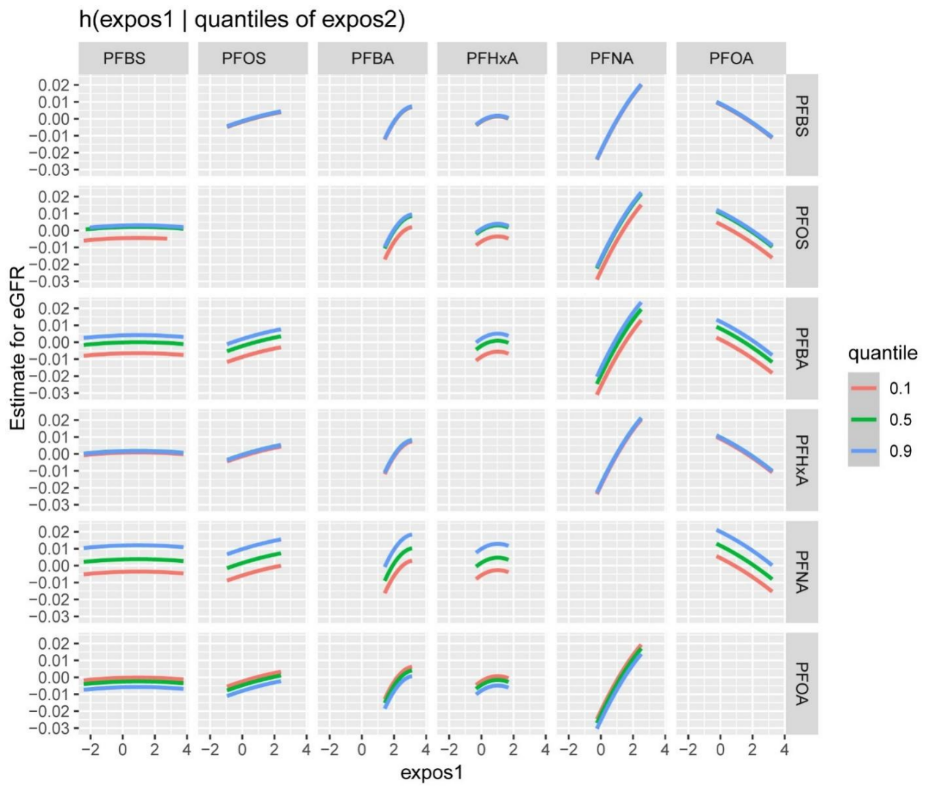


**Figure S6.** The bivariate interaction between each per- and polyfluoroalkyl substance (PFAS) in the mixture with eGFR, holding another PFAS at the 10th, 50th, and 90th percentiles and the remaining PFAS set to the median value. The Bayesian kernel machine regression (BKMR) models were adjusted for maternal age, pre-pregnant body mass index (BMI), maternal education, passive smoking, parity, delivery mode, newborn sex, birth weight, and birth height, gestational diabetes mellitus, intrauterine asphyxia, premature rupture of amniotic fluid, hypertension, and thyroid disease.


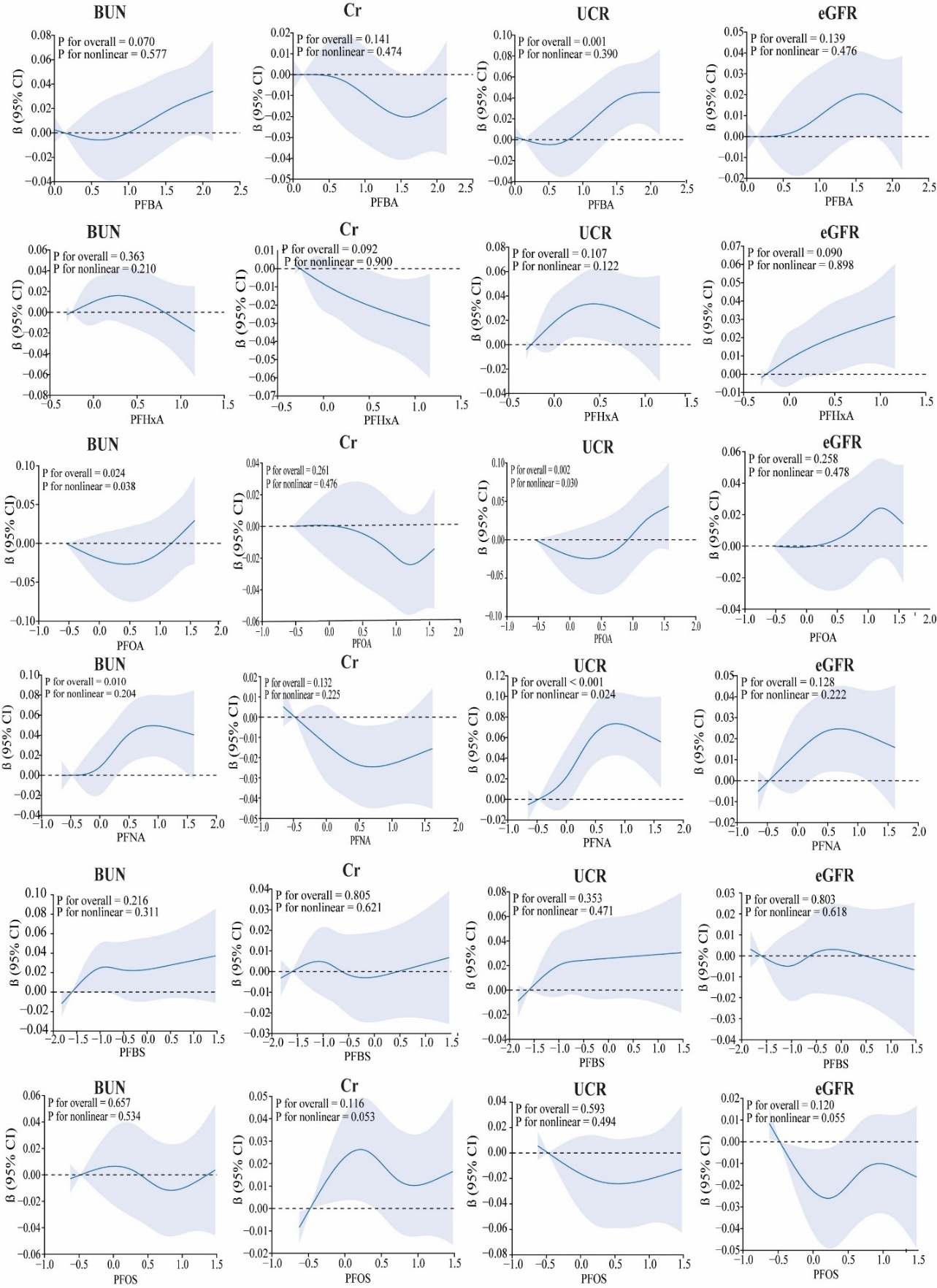


**Figure S7.** Non-linear relationship between each of the PFAS and renal function indicators. Solid lines (blue) indicate beta coefficients and shaded areas indicate 95% confidence intervals. The modes were adjusted for maternal age, pre-pregnancy body mass index (BMI), maternal education, passive smoking, parity, delivery mode, newborn sex, birth weight, birth height, gestational diabetes mellitus, intrauterine asphyxia, premature rupture of amniotic fluid, hypertension, and thyroid disease.

**
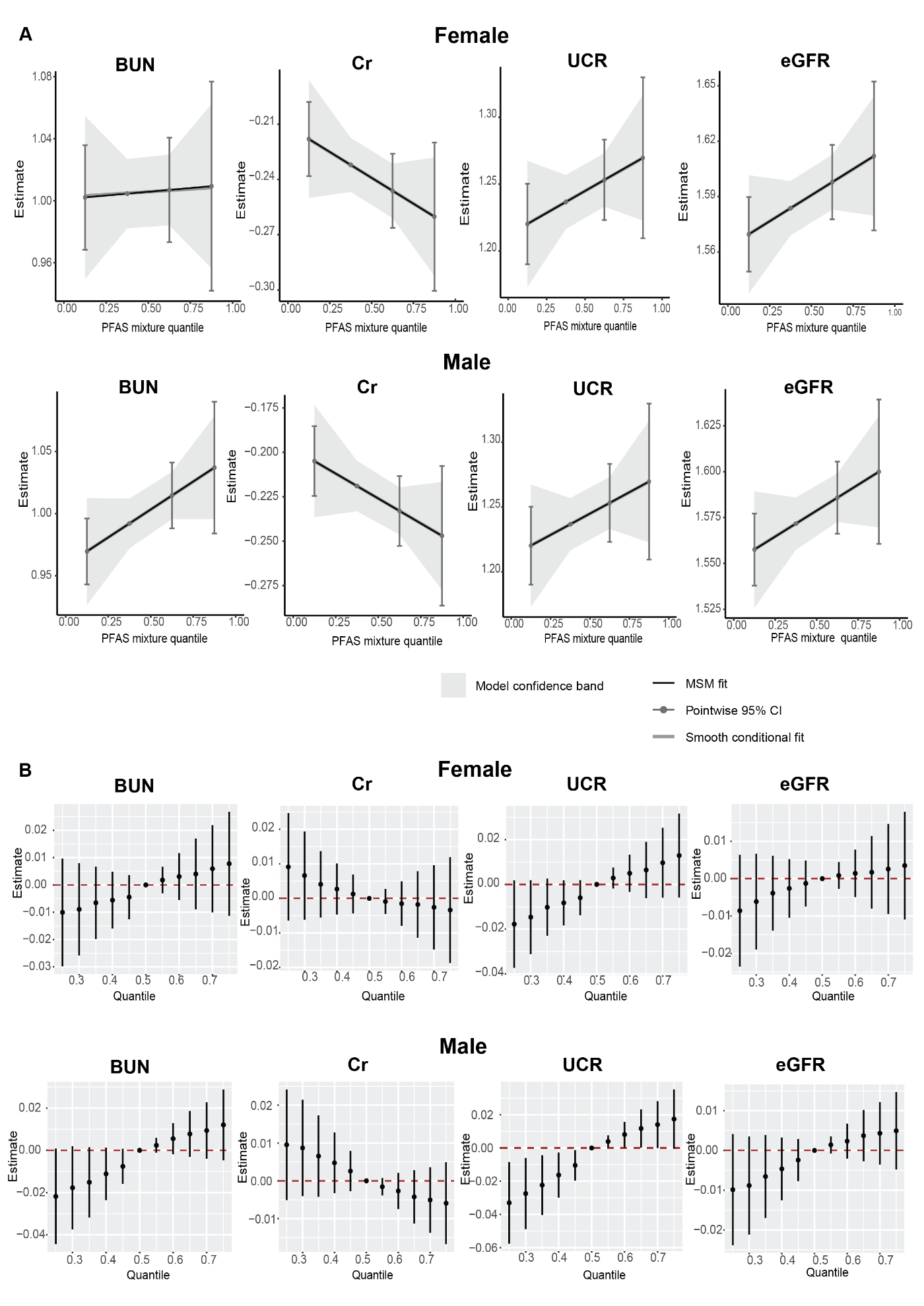
**

**Figure S8.** The overall effect of the mixture of per- and polyfluoroalkyl substances (PFAS) on renal function indicators in cord blood of newborns based on the newborn’s sex. A) The quantile g-computation regression. B) The Bayesian kernel machine regression (BKMR) models were estimated by comparing the difference when all PFAS were set at particular percentiles (25 to 75th) with their median. The models were adjusted for maternal age, pre-pregnancy body mass index (BMI), maternal education, passive smoking, parity, delivery mode, birth weight, birth height, gestational diabetes mellitus, intrauterine asphyxia, premature rupture of amniotic fluid, hypertension, and thyroid disease.

**
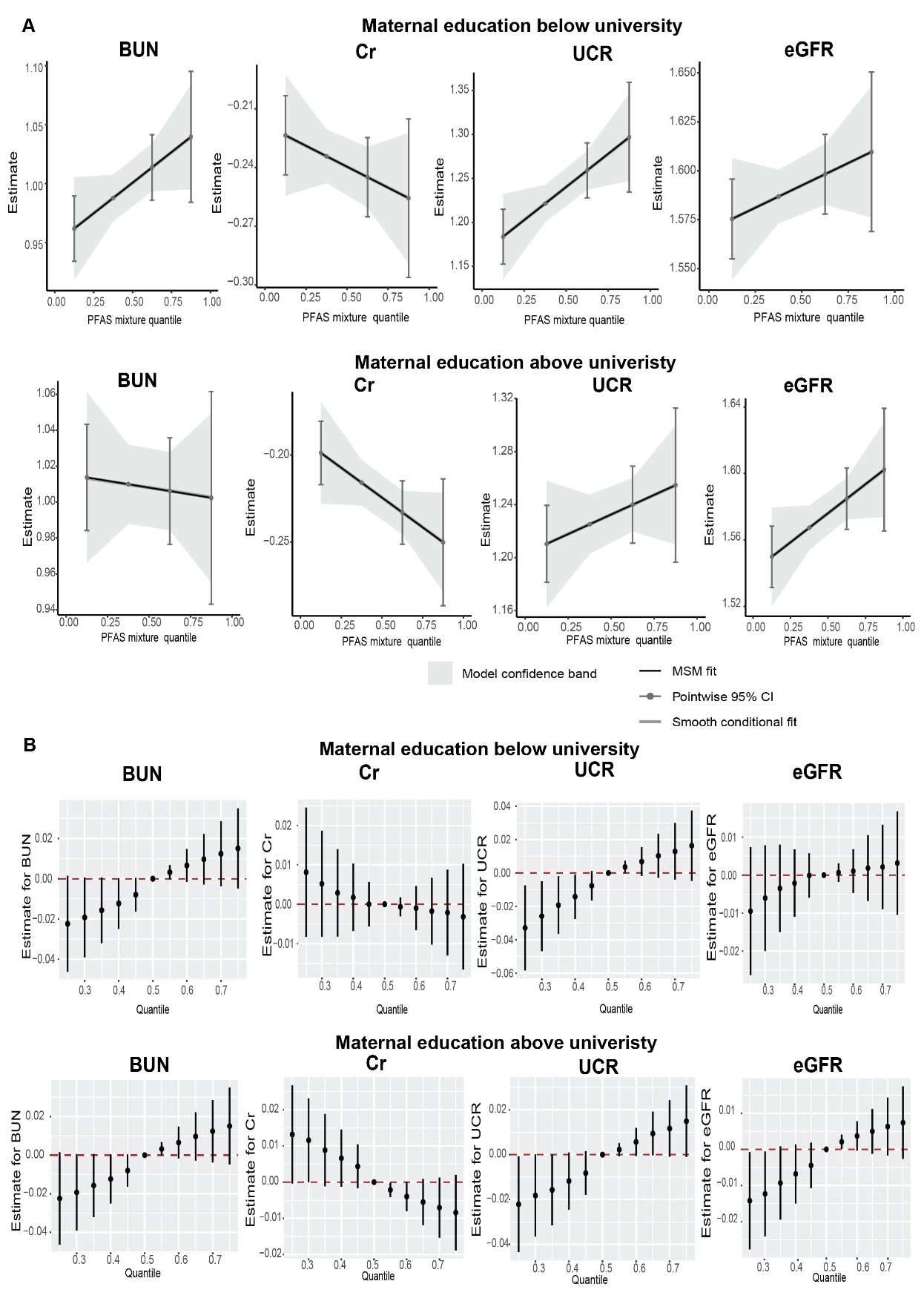
**

**Figure S9.** The overall effect of the mixture of serum per- and polyfluoroalkyl substances (PFAS) on renal function indicators in cord blood of newborns based on maternal education. A) The quantile g-computation regression. B) The Bayesian kernel machine regression (BKMR) models were estimated by comparing the difference when all PFAS were set at particular percentiles (25 to 75th) with their median. The models were adjusted for maternal age, pre-pregnancy body mass index (BMI), passive smoking, parity, delivery mode, newborn sex, birth weight, birth height, gestational diabetes mellitus, intrauterine asphyxia, premature rupture of amniotic fluid, hypertension, and thyroid disease.
